# Unraveling the Diversity of Eukaryotic Microplankton in a Large and Deep Perialpine Lake Using a High Throughput Sequencing Approach

**DOI:** 10.3389/fmicb.2020.00789

**Published:** 2020-05-07

**Authors:** Nico Salmaso, Adriano Boscaini, Massimo Pindo

**Affiliations:** Research and Innovation Centre, Fondazione Edmund Mach, San Michele all’Adige, Italy

**Keywords:** eukaryotic microplankton, protists, phytoplankton, high-throughput sequencing, amplicon sequence variants (ASVs), exact sequence variants (ESVs), deep lakes, Lake Garda

## Abstract

The structure of microbial communities, microalgae, heterotrophic protozoa and fungi contributes to characterize food webs and productivity and, from an anthropogenic point of view, the qualitative characteristics of water bodies. Traditionally, in freshwater environments many investigations have been directed to the study of pelagic microalgae (“phytoplankton”) and periphyton (i.e., photosynthetic and mixotrophic protists) through the use of light microscopy (LM). While the number of studies on bacterioplankton communities have shown a substantial increase after the advent of high-throughput sequencing (HTS) approaches, the study of the composition, structure, and spatio-temporal patterns of microbial eukaryotes in freshwater environments was much less widespread. Moreover, the understanding of the correspondence between the relative phytoplankton abundances estimated by HTS and LM is still incomplete. Taking into account these limitations, this study examined the biodiversity and seasonality of the community of eukaryotic microplankton in the epilimnetic layer of a large and deep perialpine lake (Lake Garda) using HTS. The analyses were carried out at monthly frequency during 2014 and 2015. The results highlighted the existence of a rich and well diversified community and the presence of numerous phytoplankton taxa that were never identified by LM in previous investigations. Furthermore, the relative abundances of phytoplankton estimated by HTS and LM showed a significant relationship at different taxonomic ranks. In the 2 years of investigation, the temporal development of the whole micro-eukaryotic community showed a clear non-random and comparable distribution pattern, with the main taxonomic groups coherently distributed in the individual seasons. In perspective, the results obtained in this study highlight the importance of HTS approaches in assessing biodiversity and the relative importance of the main protist groups along environmental gradients, including those caused by anthropogenic impacts (e.g., eutrophication and climate change).

## Introduction

Microbial eukaryotes are a large polyphyletic assemblage of organisms that include many groups that are more closely related to plants, fungi or animals than they are to other protists (Campbell et al., 2008). The majority of protist diversity is distinguished into a number of comprehensive monophyletic groups, which are usually referred to by the informal name “supergroups” (Guillou et al., 2013). Besides heterotrophic protists and microscopic fungi, photosynthetic and mixotrophic protists, or “microalgae,” are scattered within many supergroups along with many other protozoans, with the exception of Archaeplastida, which form a group of their own (Simpson et al., 2017).

Overall, the structure and abundance of microbial communities, microalgae, heterotrophic protozoans and fungi contribute to control productivity levels and characterize trophic webs and, from an anthropogenic perspective, the qualitative characteristics of waterbodies. Nevertheless, in freshwater environments, the majority of the investigations were historically addressed toward the study of microalgae, either pelagic (“phytoplankton”) (Padisák, 2004; Reynolds, 2006) or periphytic (Rimet et al., 2015). These two broads and loosely defined functional groups are composed of a wide variety of photosynthetic and mixotrophic organisms that show specific adaptations to different lake typologies and trophic status. Traditionally, in addition to the protist fraction, microalgae also include photosynthetic cyanobacteria (Padisák, 2004; Guiry and Guiry, 2019). In lakes, a wide variety of studies showed a clear correlation between eutrophication and the development of distinctive algal groups (such as toxigenic cyanobacteria and chlorophytes) and species (Reynolds et al., 2002; Paerl and Otten, 2013; Meriluoto et al., 2017). Further, the temporal dynamics of phytoplankton were historically investigated in many typologies of waterbodies, laying the foundation for the generalization of temporal patterns and seasonality of the main taxonomic groups determined by light microscopy (LM) (Sommer et al., 2012; De Senerpont Domis et al., 2013). Conversely, the study of non-photosynthetic protists in inland waters was mostly focused on taxonomic and broad ecological aspects of selected groups and populations (see e.g., Foissner and Berger, 1996; Wujek, 2005). In general, the knowledge of the key ecological roles of freshwater planktic microeukaryote communities has been limited by incomplete inventories of diversity (Cotterill et al., 2008; Grossmann et al., 2016).

The limitations implicit in the use of traditional microscopical determinations and identification of morphological diacritical characters has posed serious difficulties in the evaluation of the biodiversity not only in non-photosynthetic protozoans, but also in most microalgal groups (Hugerth and Andersson, 2017). Though still limited to a few applications in freshwater environments, high throughput sequencing (HTS) technologies have revealed a high level of microeukaryote biodiversity (Nolte et al., 2010; Simon et al., 2015; Cruaud et al., 2019). In the case of phytoplankton, the comparability of the relative abundance of specific taxa collected using LM and HTS remains an active field of investigation (Giner et al., 2016).

The main aim of this contribution is to characterize the biodiversity and seasonality of eukaryotic microplankton (excluding small zooplankton) in the epilimnetic layer of the large and deep perialpine Lake Garda using HTS. The work is a follow up of a recent contribution focused on the characterization of the biodiversity and seasonality of bacterial communities in the same lake (Salmaso et al., 2018a). This previous study identified a higher number of cyanobacterial taxa compared to those previously characterized by light microscopy. Heterotrophic protists in Lake Garda and in the other large lakes south of the Alps were the object of a low number of investigations (Pucciarelli et al., 2008; Asioli et al., 2009). Conversely, phytoplankton was and is one of the main biological elements included in the Italian Long Term Ecological Research (LTER^1^) network (Morabito et al., 2018; Salmaso et al., 2018b) and in the monitoring plans ruled by the Water Framework Directive (Water Framework and Directive, 2000; Pasztaleniec, 2016). Until now, phytoplankton in the southern perialpine lake district was investigated using traditional microscopy methods. Therefore, the application of HTS methods in the study of microeukaryotes is expected to amplify and complement the knowledge on heterotrophic microeukaryotes and phytoplankton in the large perialpine lakes. Specific objectives of this work include (i) the characterization and critical discussion of the composition and seasonal dynamics of planktic microeukaryotes through a 2-year study; (ii) the evaluation and comparison of the biodiversity of photosynthetic and mixotrophic protists (“phytoplankton”) evaluated by HTS and traditional microscopical methods; (iii) the quantitative evaluation of the comparability of spatial and temporal patterns of phytoplankton estimated by LM and HTS.

## Materials and Methods

### Study Site

Lake Garda is the largest Italian lake. It is located at 65 m a.s.l. and has a surface area of 368 km^2^, a volume of 49 × 10^9^ m^3^, and a maximum depth of 350 m. The lake is an important resource for irrigation, industry, drinking water supply and tourism. Owing to the great depth, complete mixing can occur only during cold winters and complete cooling of the water column. The last complete mixing of the lake was documented between 2004 and 2006. Since then, spring circulation of the water column ranged between 80 and 170 m. In the last decade, the lake underwent a slow but continuous process of oligotrophication. After 2010, total phosphorus (TP) concentrations at spring overturn in the whole water column and in the trophogenic (0–20 m) layers ranged between around 15 and 19 μg L^–1^, and 10 and 16 μg L^–1^ (Salmaso et al., 2018c, 2020).

### Environmental Variables

Samples were collected in the LTER station (45.69 N, 10.72 E) of Lake Garda, which corresponds to the deepest zone of the north-western lake (350 m). Sampling and field measurements were carried out at monthly frequency from January 2014 to October 2015 in three layers within the euphotic zone of the lake (0–2 m, 9–11 m, and 19–21 m; hereafter 1 m, 10 m, and 20 m, respectively), with a total of 34 and 30 samples collected in 2014 and 2015, respectively. Due to bad weather and dangerous lake conditions, in January 2014 samplings were carried out in the most sheltered south-eastern basin. Vertical profiles of water temperature (Temp) were carried out using a multi-parameter probe (Idronaut Ocean Seven 316Plus). Water transparency and light attenuation coefficients (*k*_d_) were measured with a Secchi disk and with a submersible irradiance sensor (LiCor 192SA), respectively. The euphotic depth was estimated as *z*_eu_ = log_e_(100) × k_d_^–1^. Nitrate nitrogen (NO_3_-N), ammonium (NH_4_-N), soluble reactive phosphorus (SRP), reactive silica (Si), Alkalinity (Alk), pH, water conductivity reported at 20°C (Cond) and dissolved oxygen (O_2_) were determined following standard methods (Cerasino and Salmaso, 2012); further details are provided in Salmaso et al. (2018a). In the analysis of data, seasons included the periods between January and March (winter), April and June (spring), July and September (Summer), and October and December (autumn).

### Phytoplankton Analyses

Phytoplankton counting (density, cell mL^–1^, and biovolume, mm^3^ m^–3^) were carried out using an inverted light microscope (Zeiss Axiovert 135) and methods described in detail by Rott et al. (2007) and Salmaso et al. (2018c). Microscopic species identification was based on the more recent monographs of the series Süßwasserflora von Mitteleuropa (Springer Spektrum) and Das Phytoplankton des Süßwassers (E. Schweizerbart’sche Verlagsbuchhandlung, Stuttgart). The classification of species into corresponding higher taxonomic ranks was based on the most recent and continuously updated literature review by Guiry and Guiry (2019). Chlorophyll-a (Chla) was estimated from spectrophotometer readings of acetone extracts following standard methods (Lorenzen, 1967).

### DNA Extraction, Library Construction and Sequencing

HTS analyses for the determination of protists were carried out on the same environmental samples filtered on GF/C filters (1.2 μm) used in the previous analyses of microbial communities using the 16S rRNA gene; the detailed procedure of DNA extraction from environmental samples is reported in Salmaso et al. (2018a). In short, DNA extraction was performed with Mo Bio PowerWater^®^ DNA Isolation Kit (MO BIO Laboratories, a QIAGEN Company, United States). All the samples showed measurable concentrations of DNA (average ± SD, 47 ± 23 ng μL^–1^), with the exclusion of two samples (June 2014, 20 m and July 2014, 1 m), which were excluded from the successive analyses.

For each individual environmental sample, total genomic DNA was subjected to PCR amplification by targeting a ∼380-bp fragment of the 18S rRNA gene variable region V4 using the specific primer set TAReuk454FWD1 (5′ CCAGCASCYGCGGTAATTCC 3′) (Stoeck et al., 2010) and TAReukREV3_modified (5′ ACTTTCGTTCTTGATYRATGA 3′) (Stoeck et al., 2010; Piredda et al., 2017) with overhang Illumina adapters. PCR amplification and library construction were performed as described in Salmaso et al. (2018a). Finally, all barcoded libraries were pooled in equimolar concentrations by qPCR in a final library and checked on a Typestation 2200 platform (Agilent Technologies, Santa Clara, CA, United States). The final library was sequenced on an lllumina^®^ MiSeq (PE300) platform (MiSeq Control Software 2.6.2.1 and Real-Time Analysis software 1.18.54).

The sequences were assigned to samples using sample-specific barcodes and saved in FASTQ formatted files. Sequences were deposited to the European Nucleotide Archive (ENA) with study accession number PRJEB36925.

### Bioinformatic and Statistical Data Analysis

Sequences were analyzed using the DADA2 package 1.12.1 (Callahan et al., 2016) in R 3.6.0 (R Core Team, 2019) and Bioconductor v. 3.9 packages (Huber et al., 2015); truncLen and trimLeft parameters were set at 275 and 230, and 20 and 21, respectively. The DADA2 error model resolves read variants (amplicon sequence variants, ASVs, also known as exact sequence variants, ESVs) that differ by as little as one nucleotide, providing exact sequence variants that replace the OTUs obtained by traditional pipelines based on the clustering of reads above a certain subjective identity (Callahan et al., 2017). Taxonomic assignment was carried out using the RDP naive Bayesian classifier method described in Wang et al. (2007) and the PR^2^ protist ribosomal reference database v. 4.11.1 with 80% minimum bootstrap confidence threshold; in the PR^2^ database, the suffix “_X” is used to indicate unknown/unnamed taxonomic levels (Guillou et al., 2013).

A total of 1248 ASVs was obtained after the application of the bioinformatic pipeline. The ASVs table, taxonomy, and environmental data were imported into the R package phyloseq 1.28.0 (McMurdie and Holmes, 2013). After removal of higher plants, higher organisms, other metazoans and unclassified taxa at the level of Division, the number of ASVs was 1073. The ASVs table was rarefied without replacement (rarefy_even_depth function in phyloseq) to the minimum number of sequences per sample (5767), obtaining a final table with 1035 ASVs. The sequences were therefore classified into three wide-ranging functional groups including “heterotrophic microplankton,” “phytoplankton” and “fungi.” The attribution of microplankton to the “phytoplankton” group followed the traditional criteria used in microscopy and phytoplankton ecology, therefore including mixotrophic species and also large heterotrophic flagellates (e.g., *Gyrodinium* and katablepharids; Wehr and Sheath, 2003; Moestrup and Calado, 2018; Guiry and Guiry, 2019), and allowing comparison with data obtained by microscopy (Reynolds, 2006; Salmaso, 2010); see Supplementary Table 1. Alpha diversity in the single samples (ASVs number and Shannon diversity) and beta-diversity (Bray and Curtis) were computed following Salmaso et al. (2018a). Differences in alpha diversity between groups of samples were estimated using the Kruskal–Wallis rank sum test (KW) and the pairwise Wilcoxon rank sum test (WR), with *p*-values adjusted using the Benjamini and Hochberg (1995) correction (R Core Team, 2019). The fraction of ASVs shared between groups of samples was visualized using Venn’s diagrams (package venn in R).

Ordination of samples based on all the eukaryotic microplankton was carried out using non-metric multidimensional scaling (NMDS) and vector fitting procedures. As for HTS data, NMDS was computed using standard parameters in the vegan package, i.e., performing a preliminary square root transformation and Wisconsin double standardization (Oksanen et al., 2018). Differences in taxa composition between groups of samples collected in different seasons and depths were tested using PERMANOVA computed on the same Bray-Curtis distance matrix used in NMDS, with 9999 bootstraps (function adonis in R vegan package; Oksanen et al., 2018). The concordance between the NMDS configurations computed using the data collected in 2014 and 2015 (i.e., correspondence between the same sampling months/depths in the 2 years) was tested by Procrustes analyses and PROTEST tests (Jackson, 1995; Legendre and Legendre, 1998). The same approach was used to compare NMDSs computed using ASVs obtained from HTS analyses and phytoplankton species biovolumes determined by LM.

Relationships among environmental variables were tested by computing Spearman ρ correlations, with *p*-values adjusted using the Benjamini and Hochberg (1995) correction. The correlation between environmental variables and the eukaryotic microplankton community was estimated by computing the Mantel test (Legendre and Legendre, 1998; Oksanen et al., 2018). The environmental distance (euclidean) matrix was computed using a set of standardized environmental variables. The microeukaryotic dissimilarity matrix was computed using the same methods used in NMDS. The significance of the statistic was evaluated by 9999 permutations of rows and columns of the dissimilarity matrix.

To evaluate the relationships between classified and unclassified ASVs at the genus level, the distribution of ASVs in selected groups (Dinoflagellata and Bacillariophyta) was carried out by mapping abundances on a phylogenetic tree built, after aligning sequences with MAFFT 7.409 (Katoh and Standley, 2013), using phyML 3.1 (Guindon et al., 2010; Salmaso et al., 2015) and the R package phyloseq; potentially poorly aligned positions and divergent regions of the alignment were checked using Gblocks (Talavera and Castresana, 2007). The DNA substitution model (GTR + I + G) was selected after calling PhyML 3.1 with the phymltest function in the R package ape. The rooted trees were built using Perkinsida and Bolidophyceae as outgroups of Dinoflagellata and Bacillariophyta, respectively.

The comparison with the phytoplankton data obtained by LM was carried out using a separate rarefied (2686 reads) HTS table including only phytoplankton taxa. To allow comparison with the current taxonomic system adopted in phytoplankton ecology and microscopy classification (Salmaso, 2010), the taxa identified by HTS were further re-classified in the corresponding phytoplankton phyla and lower taxonomic ranks using the package algaeClassify in R (Patil et al., 2019), following the classification system by Guiry and Guiry (2019). After this step, the comparison between the relative (%) abundances of phytoplankton phyla and other selected lower taxonomic groups determined using HTS (from single ASVs reads) and microscopy (from single species densities and biovolumes) was carried out by computing Spearman ρ correlations and quantile regressions with the package “quantreg” in R (Koenker, 2018). Quantile regressions are used as a robust regression method when the assumption of normality in the residuals might not be satisfied (Koenker and Bassett, 1978). The 50% quantile regression (τ = 0.50) corresponds to an estimate in which half of the observations are expected to fall below and above the regression line. Standard errors and significance of the slopes were estimated using 9999 bootstraps.

## Results

### Environmental Variability

The environmental data used in this work were analyzed in detail by Salmaso et al. (2018a). In the layer between the surface and 20 m, water temperatures were between 8 and 25°C. A strong thermal stratification, with a thermocline extending up to 25 m in 2014 and ca. 30 m in 2015, developed between May and October (Supplementary Figure 1). The Secchi disk transparency ranged between 4 and 18 m. In the cold months (November–April) and during the stratification period, the euphotic depth was between 20 m and 35 m, and 15 m and 20–25 m, respectively. The amplitude of the water mixed layer exceeded that of the z_eu_ values between October and April. SRP showed higher (ANOVA, *p* < 0.001) concentrations in winter (mean ± SE, 6.1 ± 0.7 μg L^–1^) compared to the other seasons (1.8 ± 0.5 μg L^–1^). Similarly, NO_3_-N and Si were higher (ANOVA, *p* < 0.001) in winter (320 ± 9.9 μg N L^–1^ and 0.58 ± 0.02 mg Si L^–1^) compared to the other seasons, showing minimum concentrations in summer (154 ± 16 μg N L^–1^ and 0.26 ± 0.05 mg Si L^–1^). Water temperature was positively correlated with pH (range, 7.74–8.78) (ρ = 0.54; *p* < 0.001) and negatively correlated (−0.87 < ρ < −0.36; *p* < 0.01) with O_2_ (8.7–12.9 mg L^–1^), conductivity (202–229 μS cm^–1^), alkalinity (116–163 mg L^–1^), NO_3_-N (69–383 μg N L^–1^), silica (0.05–0.74 mg Si L^–1^), SRP (0.6–19.9 μg P L^–1^), and TP (1.0–27.4 μg P L^–1^). More detailed information on the seasonal development of environmental variables were provided in Salmaso et al. (2018a). Based on the OECD (1982) thresholds, the annual averages of transparency (9.8 ± 0.9 m), TP (10.7 ± 0.5 μg P L^–1^) and Chla (2.99 ± 0.17 μg L^–1^), and the annual minimum values of transparency (4 m) and maximum of Chla (7.58 μg L^–1^) in Lake Garda were typical of oligotrophic/oligo-mesotrophic conditions.

### Community Diversity

The number of protist ASVs in the individual samples varied between 90 and 190. In both years, the number of ASVs showed significant differences in the four seasons (KW < 0.05). Compared to the winter months, the higher numbers of ASVs were observed in summer and autumn (2014; WR, *P* < 0.01) and summer (2015; WR, *P* < 0.05). These results were paralleled by higher Shannon diversity values in autumn (2014; WR, *P* < 0.10) and in summer and autumn (2015; WR, *P* < 0.01) compared to the winter months (Supplementary Figure 2). Overall, the number of total ASVs was positively linked to water temperature (*r*^2^ = 0.34, *P* < 0.001); the relationship was confirmed also considering separately heterotrophic protists (*r*^2^ = 0.34, *P* < 0.001), phytoplankton (*r*^2^ = 0.12, *P* < 0.01), and fungi (*r*^2^ = 0.20, *P* < 0.01).

Most of the protistan ASVs were shared between the three sampling depths (46%). Similarly, the fraction of ASVs shared in 2014 and 2015 was 47% (Supplementary Figure 3). Nevertheless, keeping only the most frequent taxa (543 ASVs; i.e., after removing the rarest ASVs that did not appear more than five times in at least two occasions), the number of ASVs shared between the three sampling depths and the 2 years was 80 and 78%, respectively.

### Dominant Taxonomic Groups and Taxa

**FIGURE 1:**
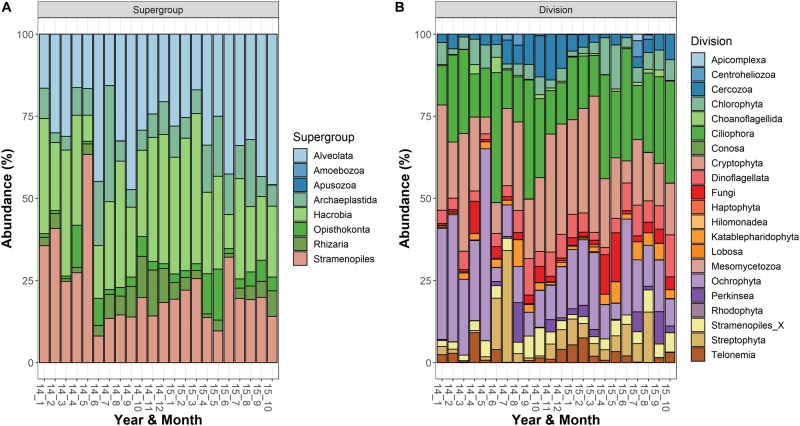
Temporal development of the microeukaryotic **(A)** Supergroups and **(B)** Divisions in Lake Garda from January 2014 to October 2015. Data refer to the averages of the three sampled layers (1, 10, and 20 m). Samples are coded by year and month. The bars report the percentage contributions on the sample totals.

The most abundant supergroups, present with a fraction of reads (averages of the three depths) greater than 10% in at least one sampling date, were Alveolata, Hacrobia, Stramenopiles, Archaeplastida, Rhizaria, and Opisthokonta (Figure 1A). Within the supergroups, the most abundant divisions in the water column (>10% in at least 1 occasion) belonged to the Alveolata (Ciliophora, 125 ASVs; Dinoflagellata, 69; and Perkinsea, 32), Stramenopiles (Ochrophyta, 222), Hacrobia (Cryptophyta, 18), Archaeplastida (Streptophyta, 15; and Chlorophyta, 63), Rhizaria (Cercozoa, 97) and Opisthokonta (Fungi, 116) (Figure 1B). Following the criteria of classifications used in the PR^2^ database, a large fraction of ASVs was classified to the family level (84%), whereas the taxonomic identifications at the genus and species levels were lower (66 and 62%, respectively). The list of taxa identified to the family level is reported in the Supplementary Table 1.

**FIGURE 2:**
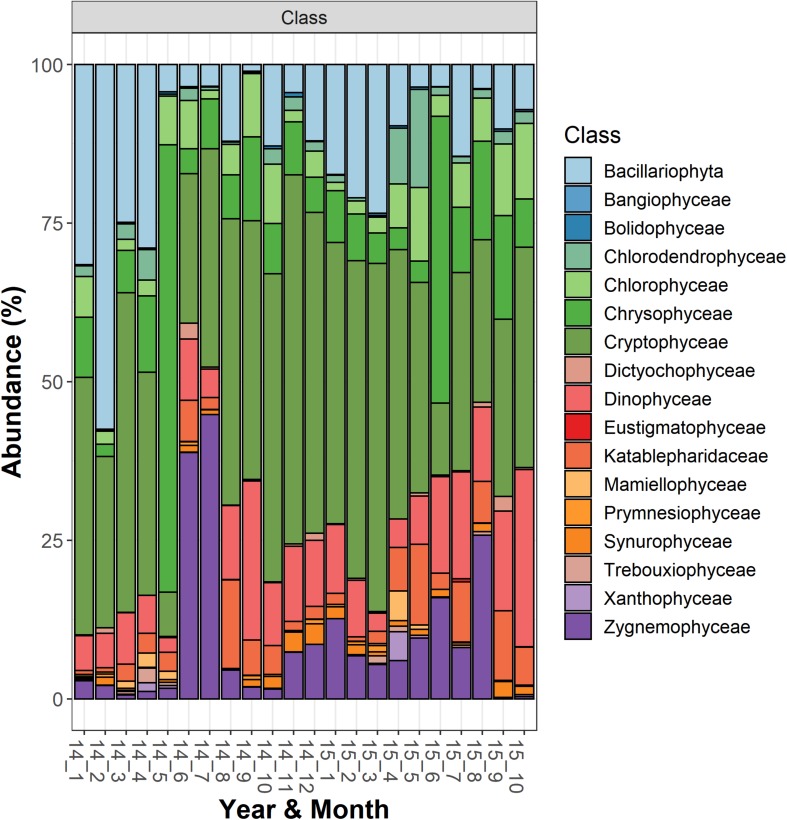
Temporal development of classes belonging to the functional group “Phytoplankton” in Lake Garda from January 2014 to October 2015. Data refer to the averages of the three sampled layers (1, 10, and 20 m). Samples are coded by year and month. The bars report the percentage contributions on the sample totals.

**FIGURE 3:**
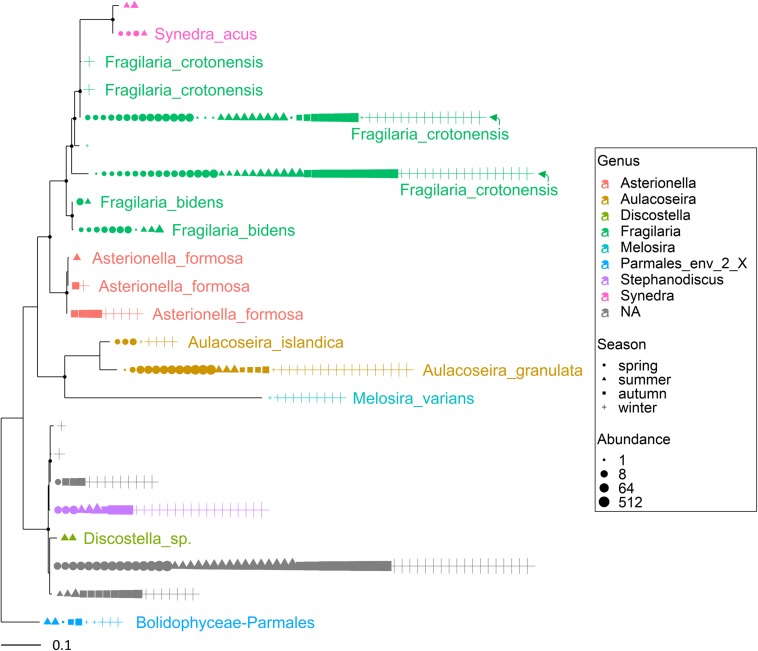
Maximum likelihood (ML) rooted topology of the class Bacillariophyta identified in Lake Garda based on alignment of 18S rRNA gene; the names of the taxa classified to the genus level (Guillou et al., 2013) are reported on the tips of the phylogenetic tree; the tree is rooted by an outgroup member of the class Bolidophyceae. Each symbol on the tip of the tree corresponds to a single sample; different symbols and colors correspond to the four seasons and genera, respectively; the size of symbols is scaled according to abundance. The small black filled circles at the nodes indicate corresponding branch support aLRT-SH-like (Anisimova and Gascuel, 2006) values > 0.85.

The most abundant microeukaryota classified at the genus level belonged to the most abundant divisions and classes (Supplementary Table 2). Among the heterotrophic protists, the Ciliophora (most of them in the class Spirotrichea) included 12 dominant genera, the most abundant being represented by *Askenasia* sp., *Rimostrombidium* spp., *Histiobalantium* sp., *Limnostrombidium* sp. and Strobilidiidae. Besides a genus belonging to the Perkinsida group, the remaining dominant taxa in the heterotrophic protists were Cercozoa and Stramenopiles (Supplementary Table 2). Overall, phytoplankton were the most represented group; the most abundant taxa were included in the class Cryptophyceae (*Cryptomonas* spp., *Plagioselmis* sp.) and classes included in the Ochrophyta, namely Chrysophyceae (*Uroglena* sp.), Bacillariophyceae (*Stephanodiscus* sp., *Fragilaria* spp., *Aulacoseira* spp.) and Synurophyceae (cf. *Synura* sp.) (Supplementary Table 2 and Figure 2). Among the wide group of “green algae,” the Zygnemophyceae were represented by *Closterium* sp. and *Mougeotia* sp., whereas Chlorophyceae and Chlorodendrophyceae by *Chlamydomonas* spp. and *Mychonastes* sp., and *Tetraselmis* sp., respectively. The class Dinophyceae (Dinoflagellata) included *Gyrodinium helveticum*, *Ceratium hirundinella*, and *Asulcocephalium miricentonis*. Once included in the cryptomonads (Wehr and Sheath, 2003), Katablepharidales were also well represented. Among Fungi, the most abundant taxon was classified among Chytridiomycetes (Rhyzophidiales). Other important taxa with a relative contribution > 1% on the total of reads in protists and classified at the family level (and therefore not included in Supplementary Table 2) comprised one Polar-centric-Mediophyceae, which, after a BLAST search, belonged to the *Cyclotella comensis/ocellata* group (i.e., the most abundant unclassified species, in gray, below *Discostella* sp. in Figure 3).

### Amplicon Sequence Variants

The most abundant genera included in Supplementary Table 2 were identified with a variable number of ASVs. A few of the taxa belonging to *Askenasia* and unnamed genera among Perkinsida_XXX, Rhyzophidiales_X and Pseudodendromonadales_XX had a large (>20) number of ASVs. In most cases, in the genera/taxa characterized by a high number of ASVs, generally only a few genotypes were dominant. For example, in the four genera considered above, only 3 individual ASVs out of 24, 30, 26, and 50 occurred with a fraction of reads greater than 10% each. The low mean similarity among sequences indicated that these groups were composed by several different species (Supplementary Table 2). This could be confirmed by the different seasonality that characterized e.g., the 3 dominant ASVs in Perkinsida, which showed three consecutive periods of growth, between July and August, and October and December/January of both years, and January and March/June 2015, respectively.

**FIGURE 4:**
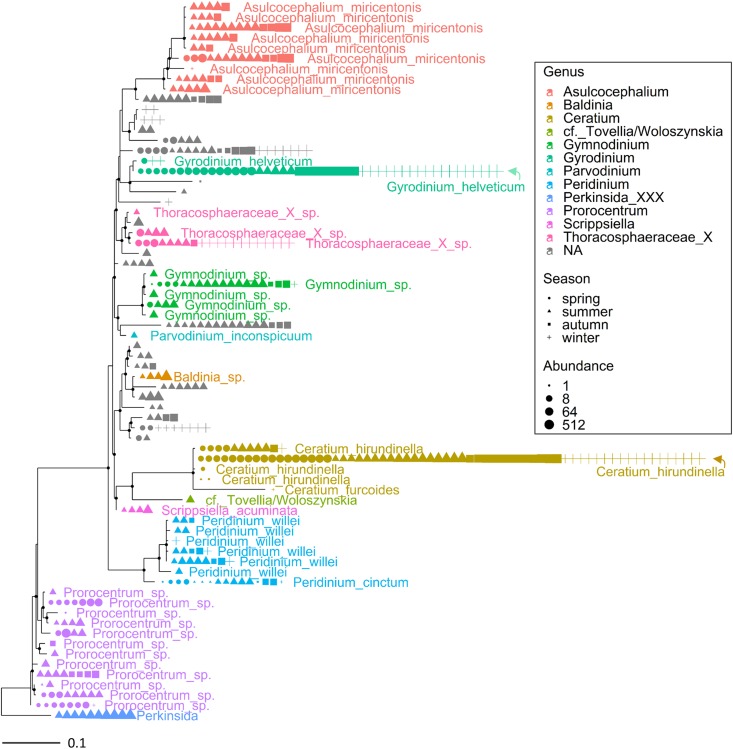
Maximum likelihood (ML) rooted topology of the class Dinophyceae identified in Lake Garda based on alignment of 18S rRNA gene; the names of the taxa classified to the genus level (Guillou et al., 2013) are reported on the tips of the phylogenetic tree; the tree is rooted by an outgroup member of the class Perkinsida. Symbols as in Figure 3.

Multiple ASVs were also identified at the species level (Figures 3, 4). Among diatoms, *Fragilaria* included 4 different ASVs belonging to *F. crotonensis*, 2 ASVs attributable to *F. bidens* and 1 ASV of unclear attribution (*F. capucina/vaucheriae*). The two most abundant *F. crotonensis* ASVs were identifiable in all months (Figure 3), with higher abundances between late winter and spring, and a significant temporal correlation between reads (ρ = 0.40, *p* < 0.01). *Asterionella formosa* was composed by 3 ASVs, mostly distributed in the autumn and winter periods. Conversely, the other diatoms classified at the species level (*Aulacoseira granulata*, *A. islandica* and *Melosira varians*) were represented by a unique ASV. In the Dinophyceae (Figure 4), *Gyrodinium helveticum* and *Ceratium hirundinella* were present with one dominant ASV. *Asulcocephalium*, which was identified as a unique species (Supplementary Table 1), had two abundant ASVs out of 9. More tenuous differences among ASVs were observed in the other genera or species (Figure 4). Among dinoflagellates, it is worth highlighting the presence of *Baldinia* (Figure 4 and Supplementary Figure 4), a species that was recognized for the first time in 2015 using microscopic and genetic (18S rDNA) analyses (see section Discussion).

Overall, the HTS analyses identified several phytoplankton genera and species that were not recognized by microscopic analyses in this work (Supplementary Table 1) and previous investigations (Salmaso, 2010; Salmaso et al., 2018c). A thorough analysis is beyond the scope of this work, but focusing the attention on the two groups considered previously (Figures 3, 4), the contribution of HTS in the evaluation of phytoplankton biodiversity is quite apparent. Besides the confirmation of dominant diatoms identified by LM, a few additional diatoms included *Fragilaria bidens* and *Discostella* sp. (Figure 3). Among dinoflagellates, additional species included *Asulcocephalium miricentonis*, *Parvodinium inconspicuum/umbonatum*, *Peridinium cinctum*, *Scrippsiella* sp. (a marine genus) as well as other taxa belonging to the Thoracosphaeraceae and Prorocentrales, and other species possibly (BLAST ca. 95%) attributable to the *Tovellia*/*Woloszynskia* group (originally classified within *Tovellia* cf. *aveirensis*) and *Ceratium* (Figure 4).

### Temporal Development of Eukaryotic Microplankton

**FIGURE 5:**
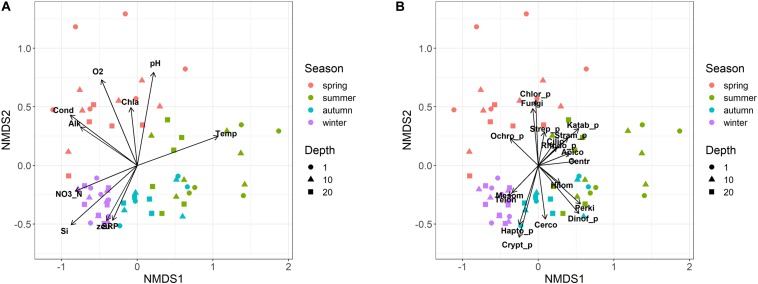
Non-metric multidimensional scaling (NMDS) ordination (stress = 0.15) of samples based on the microeukaryotic composition (ASVs). Samples are coded by season (colors) and depth (symbols). **(A)** vector fitting of significant (*P* < 0.01) environmental variables: Temp, water temperature; Cond, water conductivity; O2, dissolved oxygen; SRP, soluble reactive phosphorus; NO3_N, nitrate nitrogen; Si, reactive silica; Alk, alkalinity; zeu, euphotic depth; Chla, Chlorophyll-a. **(B)** Vector fitting (at least *P* < 0.1) of microeukaryotic divisions: Apico, Apicomplexa; Centr, Centroheliozoa; Cerco, Cercozoa; Chlor_p, Chlorophyta; Cilio, Ciliophora; Crypt_p, Cryptophyta; Dinof_p, Dinoflagellata; Fungi, Fungi; Hapto_p, Haptophyta; Hilom, Hilomonadea; Katab_p, Katablepharidophyta; Mesom, Mesomycetozoa; Ochro_p, Ochrophyta; Perki, Perkinsea; Rhodo_p, Rhodophyta; Stram_p, Stramenopiles_X; Strep_p, Streptophyta (Zygnemophyceae); Telon, Telonemia. The suffix “_p” indicates divisions totally or partly attributable to “phytoplankton.”

Eukaryotic microplankton samples showed an ordered seasonal pattern, characterized by a clear and significant clustering of samples belonging to the individual seasons in the NMDS configuration (Figure 5; PERMANOVA, *P* < 0.001). The temporal distribution of samples showed a comparable temporal pattern in both years (same sampling months/depths in 2014 and 2015; Figure 5; PROTEST test, *P* = 0.001); the repeatability of annual cycles was confirmed also after computing two separate NMDS in 2014 and 2015. The seasonal development did not show significant differences among the three sampling depths (i.e., 1, 10, and 20 m; PERMANOVA, *P* > 0.2). The regular development of the community was strongly (vector fitting, *P* < 0.01) linked to the main environmental variables (Figure 5A). The summer samples were characterized by higher water temperatures, lower concentrations of nutrients and lower euphotic depths compared to the winter samples. The spring samples were associated to high conductivity, alkalinity, pH, oxygen, and chlorophyll-a levels. Based on the whole dataset, the correlation between the environmental variables and the community structure was highly significant (Mantel statistic, *r* = 0.60, *P* < 0.001). The results were highly significant also after making the computations separately, on the three depth layers (Mantel statistic, *r* = 0.67, 0.52 and 0.51 at 1, 10, and 20 m, respectively; *P* < 0.001).

Most of the divisions belonging to heterotrophs, phytoplankton and fungi, developed in specific temporal periods, particularly between spring and summer (Figure 5B). The only divisions that showed a higher development around the winter period, or between winter and autumn, were Mesomycetozoa and Telonemia, and Haptophyta, Cryptophyta and Cercozoa, respectively. This was substantiated considering a few examples at the genus/species level. Among ciliates, *Askenasia* and *Rimostrombidium_D* showed a marked development between late spring and summer, and spring, whereas *Histiobalantium* showed a higher development in winter and between spring and early summer (Supplementary Figures 5A–C). The winter, and the winter and autumn development in the divisions Telonemia and Cercozoa were well represented by Telonemia-Group-2_X and Novel-clade-2_X (Supplementary Figures 5D,E). The development of fungi in spring was exemplified by the strict localization of Rhyzophidiales between April and May/June (Supplementary Figure 5F). Further examples for phytoplankton are reported in the next section.

### Temporal Development of Phytoplankton

**FIGURE 6:**
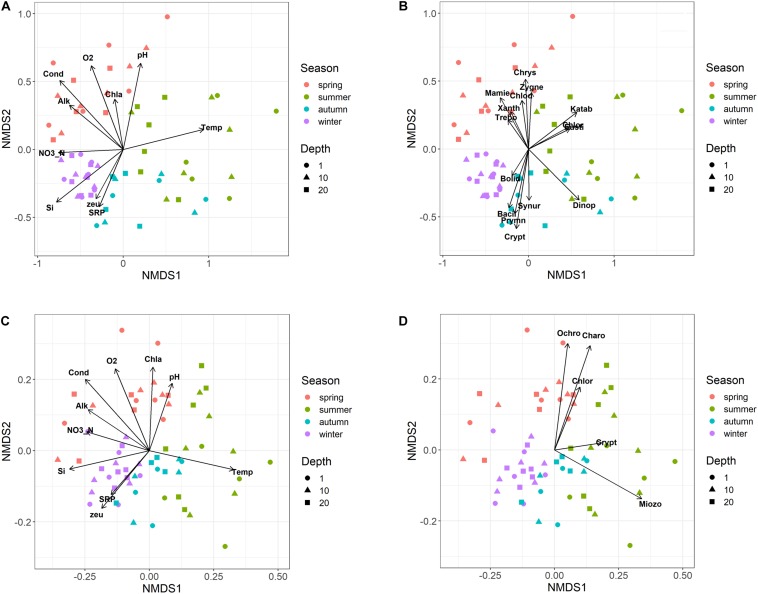
Non-metric multidimensional scaling (NMDS) of the phytoplankton community. **(A,B)** NMDS (stress = 0.17) of samples based on phytoplankton composition determined by HTS (ASVs). Samples are coded by season (colors) and depth (symbols); **(A)** vector fitting of significant environmental variables (*P* < 0.01, with the exclusion of Chla, *P* < 0.10); codes as in Figure 5A; **(B)** vector fitting of phytoplankton classes (*P* < 0.05, with the exclusion of Bolidophyceae, *P* < 0.10): Bacil, Bacillariophyta; Bolid, Bolidophyceae; Chlod, Chlorodendrophyceae; Chlor, Chlorophyceae; Chrys, Chrysophyceae; Crypt, Cryptophyceae; Dinop, Dinophyceae; Eusti, Eustigmatophyceae; Katab, Katablepharidaceae; Mamie, Mamiellophyceae; Prymn, Prymnesiophyceae; Synur, Synurophyceae; Trebo, Trebouxiophyceae; Xanth, Xanthophyceae; Zygne, Zygnemophyceae. **(C,D)** NMDS (stress = 0.21) of samples based on the biovolumes of phytoplankton species estimated by light microscopy; **(C)** vector fitting of significant environmental variables (*P* < 0.01); codes as in Figure 5A; **(D)** vector fitting of phytoplankton phyla (*P* < 0.05): Chlor, Chlorophyta; Charo, Charophyta; Ochro, Ochrophyta; Miozo, Miozoa (Dinoflagellata); Crypt, Cryptophyta; Bacillariophyta not included (*P* > 0.10).

**FIGURE 7:**
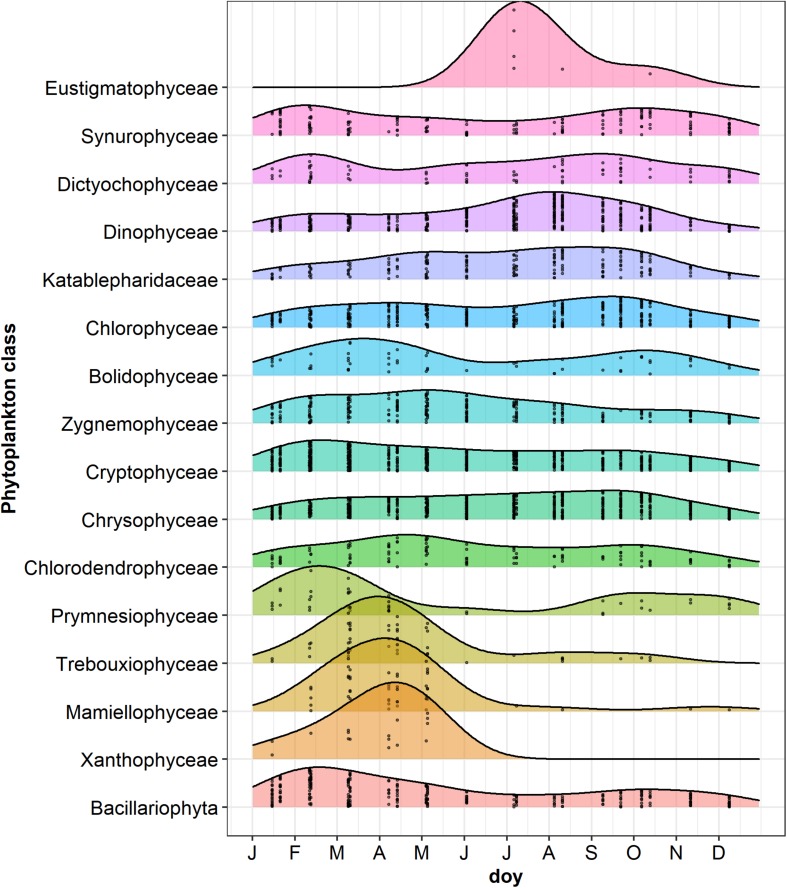
Ridgeplot showing a set of density plots of phytoplankton classes along the year. Data refer to the period from January 2014 to October 2015; jittered points indicate the occurrences of classes with a number of reads > 3 in the different sampling dates; doy, day of the year. Classes have been ordered by computing the weighted averages of the doy for every single class using the R function waps (https://github.com/hts-tools/metatools). The graph does not include Bangiophyceae (Cyanidiales), which were found only in August 2015.

The NMDS ordination of samples based on HTS phytoplankton composition (Figures 6A,B) was comparable to that obtained using the whole microplankton community (Figure 5) (PROTEST test, *P* = 0.001). Analogously, vector fitting of environmental data provided very similar results. Samples in the different seasons showed significant compositional differences (PERMANOVA, *P* = 0.001), whereas no differences were detected in the three sampled layers (PERMANOVA, *P* = 0.15) and in the temporal distribution of samples in the 2 years (PROTEST test, *P* = 0.001). As in the case of the whole microeukaryotic community, many phytoplankton classes showed a higher development during spring and summer (Figure 6B). A typical class mostly developing in the cold months was represented by Bacillariophyta, whereas the Xanthophyceae, Mamiellophyceae, Trebouxiophyceae Prymnesiophyceae and Zygnemophyceae were mostly or almost exclusively present in spring/early summer (Figure 7). Dinophyceae and Eustigmatophyceae were more frequent in the late summer and autumn months. The remaining classes were differently present in spring and autumn, and or summer months. The marked seasonality that characterized the development of phytoplankton classes was well exemplified by the temporal development of a few genera, such as *Cryptomonas* (from summer to late winter), *Ceratium* (late summer and autumn), *Asulcocephalium* (summer and autumn), *Gyrodinium* (from late autumn to late winter), *Aulacoseira* (early-mid spring), *Melosira* (late winter-early spring), and *Mougeotia* (late spring-early summer) (Supplementary Figures 6A–D,F–H). Conversely, *Baldinia* appeared only in 2015, between July and August, mostly in the upper 10 m (Supplementary Figure 6E).

The NMDS configuration obtained from the ordination of phytoplankton biovolumes determined by LM and the associated vector fitting of environmental data (Figure 6C) were fully equivalent with the results obtained using HTS data (Figure 6A) (PROTEST test, *P* = 0.001). Phytoplankton phyla showed a higher association with the spring and early summer months (Ochrophyta, Charophyta and Chlorophyta), and with mid and late summer months (Miozoa = Dinophyceae) (Figure 6D). Compared to HTS (Figure 6B), Cryptophyta in the NMDS configurations obtained from LM data showed a greater association with the summer months (Figure 6D). Moreover, owing to a broad temporal distribution, Bacillariophyceae did not show any significant association with the NMDS configuration.

**FIGURE 8:**
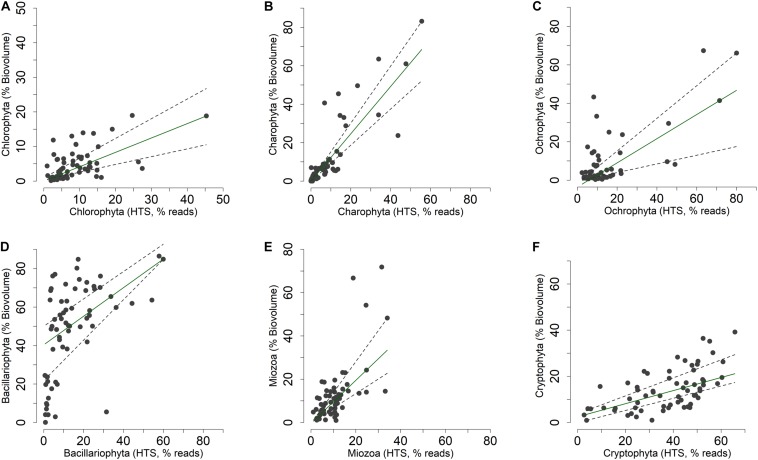
**(A–F)** Relationship between the relative abundances of the main phytoplankton phyla (Guiry and Guiry, 2019) estimated by light microscopy (% of biovolumes on the sample totals, mm^3^ m^–3^) and HTS (% of reads on the sample totals, rarefied table). Superimposed on the plots are the 0.50 (median fit: solid dark green) and 0.30 and 0.70 (dashed gray) quantile regression lines.

The concordance of the configurations obtained by HTS and microscopic data (biovolumes) suggested the existence of a relationship at least among the dominant phytoplankton groups in the two datasets. A direct comparison at predetermined higher taxonomic level of the two datasets was, however, not possible due to differences in the classifications used in the two approaches (Guillou et al., 2013; Glöckner et al., 2017; Guiry and Guiry, 2019). Nevertheless, after re-classification of phytoplankton taxonomic groups obtained in the HTS analysis into the main algal phyla usually considered in traditional phytoplankton taxonomy (Salmaso, 2010; Guiry and Guiry, 2019; Patil et al., 2019), the concordance (at least *P* < 0.05) between the two estimates (% values) was quite apparent (Figure 8 and Table 1). These results were essentially confirmed also considering the taxa agglomerated at lower taxonomic ranks, such as orders and families (Supplementary Table 3). Based on quantile regressions and biovolume values, the only groups that showed disagreement were Chlamydomonadales and Tribonematales and, partly, Synurales. In general, biovolume values provided a better concordance with the HTS abundance values (Table 1 and Supplementary Table 3). Though significant, most of the relationships between HTS, and densities and biovolumes, had slopes deviating from 1. As exemplified in Supplementary Figure 7, this was the consequence of the various methods of estimating the abundance of phytoplankton used in HTS (reads) and LM (number of cells or volume occupied by species).

**TABLE 1:** **(A)** Spearman correlation coefficients between phytoplankton abundances (% density and % biovolume) estimated by light microscopy and % HTS reads. **(B)** Quantile regressions (τ = 0.50) between % phytoplankton abundances (*y*) and % HTS reads (*x*).

(A)					(B)			

Phylum	Biovolume vs. HTS		Density vs. HTS		Biovolume vs. HTS		Density vs. HTS	
	Spearman	*P*	Spearman	*P*	SlopeQ50 (*SE*)	*P*	SlopeQ50 (*SE*)	*P*
Chlorophyta	0.54	***	0.34	**	0.42 (0.13)	**	0.65 (0.17)	***
Charophyta	0.88	***	0.89	***	1.23 (0.18)	***	0.43 (0.11)	***
Ochrophyta	0.40	**	0.29	*	0.63 (0.23)	**	0.84 (0.33)	*
Bacillariophyta	0.59	***	0.32	*	0.74 (0.30)	*	0.22 (0.21)	ns
Miozoa	0.64	***	0.71	***	0.99 (0.25)	***	0.03 (0.01)	*
Cryptophyta	0.64	***	0.54	***	0.28 (0.05)	***	0.84 (0.19)	***

SlopeQ50, regression slope; SE, standard error. Algal phyla are defined as in Guiry and Guiry (2019), following the classification system used in light microscopy. Significance codes, P: *** ≤ 0.001; ** ≤ 0.01; * ≤ 0.05; ns > 0.10.

## Discussion

This work examined the nature, extent and seasonality of the microeukaryotic community in the euphotic layer of a large perialpine lake. The HTS analyses highlighted the existence of a rich and well diversified community, and the existence of several phytoplankton taxa that were never identified in previous investigations by light microscopy. In addition, the microeukaryotic community pattern was highly consistent within individual seasons and the 2 years, providing evidence that distribution patterns were not resulting from exclusive random processes. After a brief evaluation of the constraints imposed by HTS analyses in the interpretation of data, these aspects, and implications for the study of microplankton ecology and assessment of water quality, will be addressed in the next sections.

### Constraints in the Quantitative Interpretation of HTS Data in the Study on Microeukaryotes

Inaccurate quantitative data estimates in HTS results are introduced by several factors, which include, among others, variable DNA quantity, differential DNA extraction success and different amplification rates between different species, and different quantitative estimates between different sequencers and runs (Lamb et al., 2019). Though with a large degree of uncertainty, the results of a recent meta-analysis suggested that weak quantitative relationships may exist between the biomass and number of reads (Lamb et al., 2019). Therefore, current HTS approaches quantify taxa as fractions of the sample sequence library generated by each analysis (Vandeputte et al., 2017). Comparative analyses of microplankton data should take into consideration the limits implicit in the use of relative data. As a result, attempts to evaluate functional relationships among species have the potential to introduce biases. For example, Vandeputte et al. (2017) showed how the taxonomic trade-off between two bacteria inhabiting the human microbiota, i.e., *Bacteroides* and *Prevotella*, was an artifact of relative microbiome analyses and lack of information of microbial loads, which can vary substantially between samples.

An important factor influencing abundance estimates in HTS studies is the different 18S rRNA gene copies in the cells. Compared to the prokaryotic organisms, where the 16S rDNA copy numbers are generally less than 10 (Sun et al., 2013; Stoddard et al., 2015), the range of 18S rDNA copy numbers in unicellular eukaryotes spans different orders of magnitude. In the estimates provided by Wang et al. (2017), the 18S rDNA copy numbers in dinoflagellates, diatoms and ciliates ranged from 61 and 36896, 200 and 12812, and from around 50000 to 567893, respectively. Similarly, Lofgren et al. (2019) showed that ribosomal rDNA copy numbers in fungi varied from 14 to 1442 copies. As a result, the relative abundance of 18S rDNA gene copies in different species estimated from the analysis of environmental DNA can be attributed not only to the variation in the relative abundance of microeukaryotes, but also to variation in genomic 18S rDNA copy numbers among those organisms (Mangot et al., 2013; Gong and Marchetti, 2019). The higher 18S rDNA copy numbers in the cells of ciliates could contribute to explain the large relative contribution of this group to the microeukaryotic community (Figure 1).

The effect of the variability attributable to heterogeneity in copy numbers on the abundance estimation can be attenuated by the existence of a relationship between 18S rDNA copy numbers and cell size. Godhe et al. (2008) found a highly significant positive log-log linear (power) relationship between rDNA copies and biovolumes in several selected species of dinoflagellates and diatoms. These results suggest that the relative proportions among ribotypes in microeukaryotes may reflect their proportion in biomass rather than cell abundance. Pitsch et al. (2019) found that, in ciliates, the LM biomass-based assemblage compositions had a higher similarity to 18S rDNA read numbers compared to LM cell counts. In this work, the relative abundances of most phytoplankton phyla/divisions and lower taxonomic levels estimated by HTS and LM showed a significant relationship. The association between the two methods was verified both considering cell abundances and biovolumes (biomasses). The slight better performance of biovolumes (Table 1 and Supplementary Table 3) was possibly due to the relationship between cell sizes and 18S rDNA copy numbers. It is important to note that the maximum linear dimensions of individual phytoplankton cells fall within a wide range, from ca. 0.5 to 2 μm (picoeukaryotes) to well over 100 μm (e.g., *Closterium*, thin pennate diatoms), which correspond to a variation of over 7 orders of magnitude in phytoplankton specific biovolumes (Litchman et al., 2007).

A further element that can cause overestimation in the diversity of microeukaryotes is the intragenomic heterogeneity of 18S rRNA genes (Wang et al., 2017). For example, using single-cell quantitative PCR, Gong et al. (2013) showed a significant intraindividual 18S rDNA diversity, with sequence differences primarily due to single nucleotide polymorphisms (SNPs); moreover, nucleotide diversity was positively correlated to the rDNA copy number, posing potential problems in the rDNA-based estimation of species richness. At present, considering the high sensitivity of amplicon sequence variants approaches, which can resolve biological differences of even 1 or 2 nucleotides (Callahan et al., 2016), it is not always straightforward to attribute differences to interspecific, intraspecific, or intraindividual levels (cf. Supplementary Table 2). In ciliates, Gong et al. (2013) showed that the minimum similarity among two 18S rDNA copies from the same individual was 99.1%, whereas the average similarity among copies of the same cell was 99.7 to 99.9%. Therefore, using a classical OTUs approach, the 1% similarity cut-off for clustering 18S rDNA sequences into OTUs was considered reliable to exclude intragenomic sequence variations. In ASVs, this could not however exclude the provenance of single SNPs from the same individual. Further investigations for a wider range of microeukaryotes are needed to interpret intraspecific and intraindividual 18S rDNA sequence variations. These aspects still represent and underexploited field of research (Caron and Hu, 2019).

Other methods based on OTUs clustering have been effectively used to solve the presence of sequencing errors inherent in the use of HTS approaches (Mangot et al., 2013), further highlighting that clustering and denoising strategies present important advantages and disadvantages. Nevertheless, representing a cloud of divergent sequences, *de novo* OTUs are invalid outside of the data set in which they are defined; conversely, ASVs represent exact sequences with consistent taxonomic labels, therefore allowing direct comparison of different datasets (Callahan et al., 2017). This means that ASVs generated in different studies with the same primer set can be validly merged and compared, but downstream analyses should take into account other potential methodological differences. A direct comparison of the ASVs and OTUs approaches is outside the scope of this study. Nonetheless, the efficacy of denoising approaches compared to other non-ASVs based methods has been substantiated in a number of investigations using e.g., bacterial (Glassman and Martiny, 2018; Nearing et al., 2018; Caruso et al., 2019; Prodan et al., 2020) and fungal (Pauvert et al., 2019) mock communities.

For the reasons above, the interpretation of diversity based on ASVs should take into account its multifaceted nature. ASVs do not correspond to species or even lower taxonomic levels, rather they represent different oligotypes of these same or different taxonomic levels, and even individuals (Eren et al., 2013; Salmaso, 2019). If focused on the evaluation of classical diversity estimations, downstream analyses should therefore consider unique taxa agglomerated at different taxonomic ranks, e.g., Supplementary Tables 1, 2, which however do not include all the unclassified taxa at the genus and/or family levels.

### Taxonomic Diversity

The increase in the number of heterotrophic microeukaryotes and phytoplankton ASVs in the warmer months is indicative of a greater plankton activity and production connected with increased physiological activities at higher temperatures in a thermally stable water column (Padisák, 2004). The temporal concordance among several heterotrophic protists and phytoplankton was possibly instrumental for an increase of trophic interactions among taxa, e.g., due to predation/grazing (Weisse et al., 2016). Nevertheless, considering the semiquantitative nature of abundance data in HTS analyses, the identification of functional relationships among groups and species would be unavoidably speculative (Vandeputte et al., 2017).

The scarcity of information regarding the heterotrophic protist diversity in the large and deep lakes surrounding the Alps does not allow to make any systematic comparison with previous studies. In Lake Garda, investigations were only occasional, as in 2004, after the development of a “black-spot” summer bloom caused by a ciliated protozoan, *Stentor amethystinus*, which lives symbiotically with the chlorophyte *Chlorella* (Pucciarelli et al., 2008). Since then, this species was never reported again in literature and, even in this study, representatives of *Stentor* and even members of the class Heterotrichea were no longer identified.

Conversely, the “phytoplanktonic” component within the protist community was the object of several studies carried out by light microscopy all over the large lakes north and south of the Alps (Anneville et al., 2005; Gallina et al., 2013; Salmaso et al., 2018b). Nevertheless, the limits implicit in the identification of species made by microscopy are apparent, due to the limited number of diacritical morphological features distinguishing microalgal species, especially when the qualitative and quantitative determinations are made on fixed samples. Moreover, the change in phenotype induced by environmental changes may cause individuals of the same species to be identified as dissimilar species (Luo et al., 2006). A detailed comparison of results obtained from HTS and microscopy was outside the scope of this work. Nevertheless, just focusing on two representative phytoplankton groups, i.e., diatoms and dinoflagellates, HTS allowed to confirm the presence of several dominant genera and species already and easily recognized by LM, as well as the presence of other taxa never identified by LM in previous studies. Among the dominant taxa, in the case of dinoflagellates, HTS results reflected the LM results regarding the occurrence of *Baldinia anauniensis* in Lake Garda. Using a polyphasic approach (microscopy and phylogenetic analyses), this species was identified for the first time in July 2015 during a huge surface bloom in the northern shores of the lake (harbor of Riva del Garda) (Salmaso et al., 2018c). In this work, the continued presence of *Baldinia* was documented in the upper 10 m in July and August 2015 (Supplementary Figures 4, 6E). These results showed that during the shoreline bloom, this species also developed in the pelagic photic zone. Moreover, the absence of reads in 2014 suggests that this species is not an annual member of the community.

Moving the focus to the taxa that were identified for the first time by HTS, a few were present with abundant reads in several samples, such as *Asulcocephalium miricentonis* and a taxon attributable to the Prorocentrales. *Asulcocephalium* (10–16 μm long) is difficult to distinguish by LM from other small dinoflagellates and was described only recently in Japanese freshwaters. The detection of several other closely related environmental SSU rDNA sequences suggested a worldwide distribution of this, or related, freshwater species (Takahashi et al., 2015). The Prorocentrales taxon is part of a group that mainly includes marine species and only a few freshwater taxa (Croome and Tyler, 1987; Delmail et al., 2011; Moestrup and Calado, 2018). The sequences identified in Lake Garda showed the highest% identity (BLAST, >97%) with many uncultivated strains identified in freshwater environments (Oikonomou et al., 2012; Kahn et al., 2014; Luo et al., 2017), therefore confirming the existence of a widespread taxon still waiting adequate taxonomic and ecological description. Similarly, taxonomic attributions to taxa typically observed in marine environments, such as *Scrippsiella*, requires confirmation. Using culture dependent approaches, Flaim and D’Andrea (2006) observed that an isolate microscopically identified as *Peridinium aciculiferum* was almost identical (99%, 18S rDNA LSU, 934 bp) to a strain of *Scrippsiella* sp. These authors highlighted that further studies at the ultrastructural level were needed to confirm whether the isolate was part of *Scripsiella*.

The results obtained in this work highlight the high potential of HTS in supporting the completion, amendment and refinement of microeukaryotic species lists for more robust biodiversity assessment in aquatic habitats and more reliable evaluation of water quality monitoring. In perspective, this can have a formidable impact on the update and analysis of long-term datasets, e.g., those collected within the LTER network, imposing a new level of understanding of the long-term temporal changes and patterns in the local, regional and global distribution of freshwater planktic organisms. On the other side, biodiversity assessment using modern marker gene amplification approaches is severely limited both by the short length of reads obtained by present technologies, and by the incompleteness of genetic databases, which are still fed by information obtained through isolation and cultivation approaches. In Figures 3, 4, the gray branches corresponded both to reads with ambiguous classification due to the poor discriminant power of short reads, and to taxa that did not show any correspondence below the order or family rank. Further, the similarity of short 18S rDNA sequences between non-closely related species represents serious challenges in the classification of species (Escobar-Zepeda et al., 2018), and the use of different hypervariable regions and primers can impact alpha diversity estimates (Guillou et al., 2013; Tragin et al., 2018). When a particular group of microeukaryotes are to be investigated, short regions with higher resolution can be considered, included *rbcL* for diatoms (Rimet et al., 2018), and plastidial 16S rRNA genes of photosynthetic eukaryotes (Decelle et al., 2015; Needham and Fuhrman, 2016). Therefore, the number of taxa and their identification at the genus and/or species level can be considered indicative, pending further confirmation (possibly through isolation and analysis of the most representative taxa).

### Seasonal Dynamics and Functions of Heterotrophic Microeukaryotes

The temporal development of the microeukaryotes showed a strong seasonality at different taxonomic levels, from supergroups, divisions and classes (Figures 1, 2, 7), to individual taxa (Supplementary Figures 5, 6). Microeukaryotes showed a strong link with the main physical and chemical variables controlling cyclical environmental gradients that characterize large lakes in temperate regions (Salmaso et al., 2018b). These results demonstrated the strong deterministic control of ecological processes in the assembly of different microeukaryotic communities adapted to different environmental and ecological conditions. From a wider perspective, and contrasting with former assumption of unlimited dispersal, these observations are in agreement with the more recent studies based on massive amplification of DNA markers by HTS, which demonstrated that many protists have actually markedly restricted distributions (Khomich et al., 2017). Using a large-scale molecular sampling based on standardized HTS methods, Grossmann et al. (2016) showed that limited dispersal and distribution in protists differed by habitat type and taxonomic group, without different patterns of distribution between rare and abundant taxa.

Protistan assemblages can rapidly change in short-term periods (Vigil et al., 2009; Mangot et al., 2013). Nevertheless, the equivalent and predictable seasonal pattern in the temporal development of protists in the two studied years is indicative that, in large and deep lakes, a monthly sampling is sufficient to observe reproducible pattern among the planktic community. This can be considered a distinctive trait of large and deep lakes with high renewal time. These waterbodies have the tendency to operate as large inertial systems, minimizing the effects of external disturbances (Salmaso et al., 2018c).

Among the heterotrophic microeukaryotes, ciliates were the most abundant group. Representatives of this division have a wide range of trophic lifestyles, spanning from particle feeding to symbiosis and parasitism. Overall, though not exclusively (e.g., *Histiobalantium*), the most abundant ciliates were mostly associated with higher temperatures, from mid spring to autumn, such as *Askenasia* and *Rimostrombidium*. *Askenasia* is a mixotrophic/omnivorous and carnivore ciliate predating on other ciliates and possibly dinoflagellates (Earland and Montagnes, 2002). In a nutrient-rich temperate estuary, Haraguchi et al. (2018) showed that the high grazing rates during summer was associated with high biomass of *Askenasia*. *Rimostrombidium* is a ciliate feeding on particles. Posch et al. (2015) demonstrated that this species in Lake Zurich acted as a primary consumer of cryptomonads. In this work, this was supported by the negative association (ρ = −0.49, *p* < 0.001) between the order Choreotrichida and the class Cryptophyceae (cf. Supplementary Table 2). *Histiobalantium*, which developed in the spring months and, partly, winter, is a diffusion feeding ciliate, which can ingest small algae such as cryptophytes (Müller and Schlegel, 1999). Other important taxa included *Limnostrombidium* (mostly spring and autumn), a mixotrophic coarse filter-feeding taxon (Macek et al., 2006), and *Tintinnidium*, a phytoplankton-consuming ciliate (e.g., ingesting *Stephanodiscus*; Meier and Reck, 1994). With a different ecological habit, after a free-swimming stage, *Vorticella* attach to substrates (soil, mud, plant roots, living substrata) by stalks. In Lake Garda and other lakes, *Vorticella* was found attached to many filaments of the cyanobacterium *Dolichospermum lemmermannii* (Canter et al., 1992; Salmaso et al., 2015). The buoyancy provided by the aerotopes of the host was sufficient to keep in suspension both the cyanobacterium and the ciliate. With this association, *Vorticella* is able to feed on the picoplanktonic organisms in the epilimnetic waters (Canter et al., 1992).

Besides ciliates, other heterotrophic protists were represented by members of the Perkinsida. This group is widely distributed in marine environments, and only recently it was increasingly reported also in freshwater environments (Bråte et al., 2010b; Ortiz-Álvarez et al., 2018). Perkinsida can be parasites of a variety of aquatic organisms, including algae, bivalves, fish and amphibians. Similarly, beside marine environments, Bråte et al. (2010a) demonstrated a wide presence of *Telonemia*, a group feeding on bacteria and phytoplankton, in different lakes. Cercozoans taxa include amoeboids and flagellates that feed using filose pseudopods.

Among fungi, Chytridiomycota (Chytrids) are unicellular and swim by undulating a single flagellum (Longcore and Simmons, 2012). The family Chytridiomycetes is known to contain a number of parasitic species infecting e.g., amphibians.

### Seasonal Dynamics of Phytoplankton

The understanding of the factors controlling the seasonality of microeukaryotes was widely investigated for many of the taxa belonging to the functional group of “phytoplankton”. The fraction of this group with dimensions greater than 2–4 μm was the object of countless ecological investigations based on microscopical observations. These available studies allow synthesis of information in a consistent framework (Harris, 1986; Munawar and Talling, 1986; Padisák, 2004; Reynolds, 2006; Sommer et al., 2012).

Most of the results obtained in this work were consistent with the results based on microscopic observations, and with the community patterns obtained in previous investigations in Lake Garda (Salmaso, 2010; Salmaso et al., 2018c) or synthetized in more comprehensive works (Sommer et al., 2012; De Senerpont Domis et al., 2013). Moreover, the comparison of HTS phytoplankton data with the corresponding normalized data obtained by microscopy showed a significant concordance. Comparable results included: the localization of large filamentous Bacillariophyta (*Aulacoseira* and *Melosira*) in spring and tabular colonies (*Fragilaria*) in spring and autumn; the development of filamentous Xanthophyceae (*Tribonema*) in spring, and Zygnemophyceae (*Closterium*) in late autumn/winter, and spring and early summer (*Mougeotia*); the year round presence of Chrysophyceae and Cryptophyceae; the increase of Chlorophyceae (Chlamydomonadales and Sphaeropleales) from spring to autumn; and the presence of Dinophyceae mostly in the summer months (e.g., *Ceratium* and *Peridinium*).

These temporal patterns are explained with specific environmental requirements and tolerances (Sandgren, 1988; Reynolds et al., 2002; Reynolds, 2006; Sommer et al., 2012). For example, the large and heavy siliceous Bacillariophyta require sufficient levels of turbulence to remain in suspension. This requirement is fulfilled in late winter and spring, while in summer the thermal stratification causes a rapid sinking of the large diatoms in the hypolimnetic waters. To a lesser extent, similar requirements are met also by the large *Mougeotia*, *Closterium*, and *Tribonema*, with species that respond positively to moderate water mixing and illumination (Tapolczai et al., 2015). Compared to other obligate phototrophic microalgae, the mixotrophic character of Chrysophyceae, Cryptophyceae and Dinophyceae provides an alternative strategy for these algal groups at low inorganic nutrient concentrations, especially during periods of low water mixing, when the uptake of nutrients is limited by diffusion into the cell (Sandgren, 1988; Ward et al., 2011). The Sphaeropleales (“Chlorococcales”) is a diversified group that respond positively to the increasing thermal stability by adopting a wide range of morphological adaptations to contrast large sinking losses, such as small size and mucilage formation (Reynolds, 2006, 2007).

The comparison of phytoplankton data obtained using microscopy and HTS is made difficult also by the use of different classification systems which are not directly comparable. Moreover, a number of organisms identified by HTS does not find correspondence in the data obtained by microscopy. Among others, this is the case of picoeukaryotic algae (e.g., Mamiellophyceae), the numerous simple unicellular flagellated microalgae attributable to the Trebouxiophyceae and Chlorophyceae and in general all the other groups difficult to identify by LM (e.g., Xanthophyta).

The significant correspondence between the relative phytoplankton abundances estimated by HTS and LM is a promising element for the comparability of data and their interpretation based on the two approaches. In principle, this correspondence could not be required if the evaluation of HTS data is based on standardized and consistent methods. Yet, this would require a completely new evaluation of relationships among algal groups in different environmental gradients and at different taxonomic levels. Considering that most of the ecology of phytoplankton and interpretation of their distribution along environmental gradients is based on LM, the comparability of the two approaches could facilitate the use of interpretative criteria regarding the distribution and seasonality of phytoplankton recognized with the traditional approaches (e.g., Harris, 1986; Padisák, 2004; Reynolds, 1997, 2006). This aspect was previously shown and discussed by Medinger et al. (2010) and Giner et al. (2016) and should be taken into account in the interpretation of results based on the two methods.

## Conclusion

Despite limits implicit in the quantitative estimation of microeukaryotes abundances, the application of HTS allowed obtaining a more complete picture of the microeukaryotic diversity in a large and deep perialpine lake. The contribution of HTS was particularly apparent when considering the comparison with the data of phytoplankton estimated by microscopy. HTS confirmed the dominant species determined by LM and highlighted the presence of several other species, including a few taxa not described to lower taxonomic levels in the datasets. Further, the relative abundances of phytoplankton phyla/divisions estimated by HTS and the biovolumes obtained by LM showed a significant relationship, providing perspectives in the use of HTS approaches in the evaluation of biodiversity and relative importance of major phytoplankton groups along environmental gradients, including eutrophication and other anthropogenic impacts. This view is further supported by the strong deterministic role of environmental and biotic variables in the assembly of different microeukaryotic assemblages and populations adapted to different ecological conditions along the temporal gradient. Finally, HTS are contributing to change the traditional concept of “phytoplankton,” providing a more comprehensive picture of both traditional phytoplankton groups determined by LM and the whole prokaryotic and eukaryotic planktic community.

## Data Availability Statement

The datasets generated for this study has been deposited to the European Nucleotide Archive (ENA) with study accession number PRJEB36925.

## Author Contributions

NS designed the overall work, performed the data analysis and interpretation, and wrote the manuscript. AB collected the data, contributed to the laboratory analyses, and revised the manuscript. MP contributed to the design of the experimental work, performed sequencing analyses, and revised the manuscript. All authors have approved and corrected the final version of the manuscript.

## Conflict of Interest

The authors declare that the research was conducted in the absence of any commercial or financial relationships that could be construed as a potential conflict of interest.

## Footnotes

1 http://www.lteritalia.it/
