# Supplementary material for: Unraveling the Diversity of Eukaryotic Microplankton in a Large and Deep Perialpine Lake Using a High Throughput Sequencing Approach
Source: Front Microbiol. 2020 May 7;11:789. doi: 10.3389/fmicb.2020.00789 (PMC7221148; doi:10.3389/fmicb.2020.00789)
Supplement: Supplementary file 8 [file Data_Sheet_1.PDF]

Supplementary Table 1 Microeukaryotic taxa identified at least at the family level in Lake Garda between 2014 and 2015. A) Heterotrophic protists; B) "Phytoplankton"; C) Fungi
In B), genera identified by using light microscopy in this investigation and in previous LTER analyses (see text) are indicated with the suffix "*".

A) Protists (excluding "Phytoplankton")

Supergroup	Division	Class	Order	Family	Genus	Species
Alveolata	Apicomplexa	Apicomplexa_X	Colpodellidae	Colpodellidae_X	Colpodella	Colpodella_edax
	Ciliophora	Colpodea	Colpodea_X	Colpodida	Colpoda	Colpoda_steinii
	Ciliophora	Colpodea	Colpodea_X	Cyrtolophosidida	Cyrtolophosis	
	Ciliophora	Colpodea	Colpodea_X	Cyrtolophosidida	Pseudocyrtolophosis	Pseudocyrtolophosis_sp.
	Ciliophora	Colpodea	Colpodea_X	Platyophryida	Woodruffides	Woodruffides_metabolicus
	Ciliophora	CONTH_4	CONTH_4_X	CONTH_4_XX	CONTH_4_XXX	CONTH_4_XXX_sp.
	Ciliophora	CONTH_5	CONTH_5_X	CONTH_5_XX	CONTH_5_XXX	CONTH_5_XXX_sp.
	Ciliophora	CONTH_7	CONTH_7_X	CONTH_7_XX	CONTH_7_XXX	CONTH_7_XXX_sp.
	Ciliophora	CONThreeP	CONThreeP_X	CONThreeP_XX	Askenasia	Askenasia_sp.
	Ciliophora	CONThreeP	CONThreeP_X	CONThreeP_XX	CONThreeP_XXX	CONThreeP_XXX_sp.
	Ciliophora	CONThreeP	CONThreeP_X	Urotrichidae	Urotricha	Urotricha_sp.
	Ciliophora	Oligohymenophorea	Hymenostomatia	Ophryoglenida		
	Ciliophora	Oligohymenophorea	Hymenostomatia	Tetrahymenida		
	Ciliophora	Oligohymenophorea	Hymenostomatia	Tetrahymenida	Tetrahymenida_X	Tetrahymenida_X_sp.
	Ciliophora	Oligohymenophorea	OLIGO4	OLIGO4_X	OLIGO4_XX	OLIGO4_XX_sp.
	Ciliophora	Oligohymenophorea	Oligohymenophorea_X	Oligohymenophorea_XX	Oligohymenophorea_XXX	Oligohymenophorea_XXX_sp.
	Ciliophora	Oligohymenophorea	Peniculia	Peniculida	Peniculida_X	Peniculida_X_sp.
	Ciliophora	Oligohymenophorea	Peritrichia_2	Sessilida		
	Ciliophora	Oligohymenophorea	Peritrichia_2	Sessilida	Vorticella	
	Ciliophora	Oligohymenophorea	Peritrichia_2	Sessilida	Vorticella	Vorticella_aequilata
	Ciliophora	Oligohymenophorea	Peritrichia_2	Sessilida	Vorticella	Vorticella_microstoma
	Ciliophora	Oligohymenophorea	Peritrichia_2	Vaginicolidae		
	Ciliophora	Oligohymenophorea	Peritrichia_2	Vaginicolidae	Vaginicolidae_X	Vaginicolidae_X_sp.
	Ciliophora	Oligohymenophorea	Scuticociliatia_1	Philasterida		
	Ciliophora	Oligohymenophorea	Scuticociliatia_2	Histiobalantiidae	Histiobalantium	Histiobalantium_sp.
	Ciliophora	Oligohymenophorea	Scuticociliatia_2	Histiobalantiidae	Histiobalantium	
	Ciliophora	Phyllopharyngea	Cyrtophoria_4	Chilodonellidae	Chilodonella	
	Ciliophora	Phyllopharyngea	Cyrtophoria_4	Chilodonellidae	Trithigmostoma	Trithigmostoma_cucullulus
	Ciliophora	Spirotrichea	Choreotrichida	Strobilidiidae	Strobilidiidae_X	Strobilidiidae_X_sp.
	Ciliophora	Spirotrichea	Choreotrichida	Strobilidiidae_A	Rimostrombidium_A	Rimostrombidium_A_sp.
	Ciliophora	Spirotrichea	Choreotrichida	Strobilidiidae_B	Strobilidiidae_B_X	Strobilidiidae_B_X_sp.
	Ciliophora	Spirotrichea	Choreotrichida	Strobilidiidae_C	Strobilidiidae_C_X	Strobilidiidae_C_X_sp.
	Ciliophora	Spirotrichea	Choreotrichida	Strobilidiidae_D	Rimostrombidium_D	Rimostrombidium_D_sp.
	Ciliophora	Spirotrichea	Choreotrichida	Strobilidiidae_E	Strobilidium_E	Strobilidium_E_sp.
	Ciliophora	Spirotrichea	Choreotrichida	Strobilidiidae_G	Strobilidiidae_G_X	Strobilidiidae_G_X_sp.
	Ciliophora	Spirotrichea	Choreotrichida	Strobilidiidae_J	Strobilidiidae_J_X	Strobilidiidae_J_X_sp.
	Ciliophora	Spirotrichea	Hypotrichia	Halteriidae	Halteriidae_X	Halteriidae_X_sp.
	Ciliophora	Spirotrichea	Hypotrichia	Halteriidae		
	Ciliophora	Spirotrichea	Hypotrichia	Hypotrichia_X	Hypotrichia_XX	Hypotrichia_XX_sp.
	Ciliophora	Spirotrichea	Strombidiida	Pelagostrombidiidae	Limnostrombidium	Limnostrombidium_sp.
	Ciliophora	Spirotrichea	Strombidiida_A	Strombidiida_A_X	Strombidiida_A_XX	Strombidiida_A_XX_sp.
	Ciliophora	Spirotrichea	Tintinnida	TIN_03		
	Ciliophora	Spirotrichea	Tintinnida	Tintinnidiidae	Tintinnidium	Tintinnidium_fluviatile
	Ciliophora	Spirotrichea	Tintinnida	Tintinnidiidae	Tintinnidium	Tintinnidium_sp.
	Perkinsea	Perkinsida	Perkinsida_X	Parvilucifera-group	Parvilucifera-group_X	Parvilucifera-group_X_sp.
	Perkinsea	Perkinsida	Perkinsida_X	Perkinsida_XX		
	Perkinsea	Perkinsida	Perkinsida_X	Perkinsida_XX	Perkinsida_XXX	Perkinsida_XXX_sp.
Amoebozoa	Conosa	Variosea	ATCC50593-Flamella-WIM80-lineage	Flamella-lineage	Flamella	Flamella_arnhemensis
	Conosa	Variosea	Variosea_X	Protosteliids	Protostelium	Protostelium_sp.
	Lobosa	Tubulinea	Echinamoebida	Vermamoebidae	Hartmannella	Hartmannella_vermiformis
Apusozoa	Hilomonadea	Planomonadida	Planomonadidae	Planomonadidae_Group-1	Ancyromonas	
	Hilomonadea	Planomonadida	Planomonadidae	Planomonadidae_Group-1		
Hacrobia	Centroheliozoa	Centroheliozoa_X	Centroheliozoa_XX	Centroheliozoa_XXX	Centroheliozoa_XXXX	Centroheliozoa_XXXX_sp.
	Centroheliozoa	Centroheliozoa_X	Pterocystida	Pterocystidae	Pterocystidae_X	Pterocystidae_X_sp.
	Centroheliozoa	Centroheliozoa_X	Pterocystida	Pterocystidae	Pterocystis	Pterocystis_foliacea
	Telonemia	Telonemia_X	Telonemia_XX	Telonemia-Group-1		
	Telonemia	Telonemia_X	Telonemia_XX	Telonemia-Group-2	Telonemia-Group-2_X	Telonemia-Group-2_X_sp.
Opisthokonta	Choanoflagellida	Choanoflagellatea	Choanoflagellatea_X	Choanoflagellatea_X_Group_L	Choanoflagellatea_X_Group_L_X	Choanoflagellatea_X_Group_L_X_sp.
	Choanoflagellida	Choanoflagellatea	Choanoflagellatea_X	Choanoflagellatea_X_Group_P	Choanoflagellatea_X_Group_P_X	Choanoflagellatea_X_Group_P_X_sp.
	Choanoflagellida	Choanoflagellatea	Craspedida	Monosigidae_Group_A	Codosiga	Codosiga_botrytis
	Choanoflagellida	Choanoflagellatea	Craspedida	Monosigidae_Group_O	Monosigidae_Group_O_X	Monosigidae_Group_O_X_sp.
	Choanoflagellida	Choanoflagellatea	Craspedida	Salpingoecidae_Group_C1	Salpingoeca_C1	Salpingoeca_fusiformis
	Choanoflagellida	Choanoflagellatea	Craspedida	Salpingoecidae_Group_C1	Salpingoecidae_Group_C1_X	Salpingoecidae_Group_C1_X_sp.
	Choanoflagellida	Choanoflagellida_X	Choanoflagellida_XX	Clade-2	Sphaeroeca	
	Choanoflagellida	Choanoflagellida_X	Choanoflagellida_XX	Clade-2	Sphaeroeca	Sphaeroeca_leprechaunica
	Mesomycetozoa	Ichthyosporea	Ichthyosphonida	Abeoformidae_Group_MAIP_2	Abeoformidae_Group_MAIP_2_X	Abeoformidae_Group_MAIP_2_X_sp.
	Mesomycetozoa	Ichthyosporea	Ichthyosphonida	Pseudoperkinsidae	Pseudoperkinsidae_X	Pseudoperkinsidae_X_sp.
Rhizaria	Cercozoa	Cercozoa_X	Cercozoa_XX	Cercozoa_XXX	Cercozoa_XXXX	Cercozoa_XXXX_sp.
	Cercozoa	Endomyxa	Vampyrellida	Leptophryidae	Leptophryidae_X	Leptophryidae_X_sp.
	Cercozoa	Filosa-Granofilosea	Filosa-Granofilosea_X	Novel-Gran-234	Novel-Gran-234_X	Novel-Gran-234_X_sp.
	Cercozoa	Filosa-Imbricatea	Filosa-Imbricatea_X	Novel-clade-2	Novel-clade-2_X	Novel-clade-2_X_sp.
	Cercozoa	Filosa-Imbricatea	Spongomonadida	Spongomonadidae	Spongomonadidae_X	Spongomonadidae_X_sp.
	Cercozoa	Filosa-Imbricatea	Spongomonadida	Spongomonadidae	Spongomonas	
	Cercozoa	Filosa-Imbricatea	Spongomonadida	Spongomonadidae	Spongomonas	Spongomonas_solitaria
	Cercozoa	Filosa-Sarcomonadea	Cercomonadida	Cercomonadidae	Cercomonas	Cercomonas_sp.
	Cercozoa	Filosa-Sarcomonadea	Cercomonadida	Paracercomonadidae	Paracercomonas	
	Cercozoa	Filosa-Sarcomonadea	Glissomonadida	Bodomorphidae	Bodomorpha	Bodomorpha_minima
	Cercozoa	Filosa-Sarcomonadea	Glissomonadida	Bodomorphidae	Bodomorpha	Bodomorpha_prolixa
	Cercozoa	Filosa-Sarcomonadea	Glissomonadida	Sandonidae		
	Cercozoa	Filosa-Sarcomonadea	Glissomonadida	Sandonidae	Sandona	Sandona_sp.
	Cercozoa	Filosa-Sarcomonadea	Pansomonadida	Agitatidae	Agitatidae_X	Agitatidae_X_sp.
	Cercozoa	Filosa-Sarcomonadea	Pansomonadida	Pansomonadida_X	Pansomonadida_XX	Pansomonadida_XX_sp.
	Cercozoa	Filosa-Thecofilosea	Cryomonadida	Cryomonadida_X	Cryomonadida_XX	Cryomonadida_XX_sp.
	Cercozoa	Filosa-Thecofilosea	Cryomonadida	Cryothecomonas-lineage		
	Cercozoa	Filosa-Thecofilosea	Cryomonadida	Protaspa-lineage	Protaspa	Protaspa_grandis
	Cercozoa	Filosa-Thecofilosea	Cryomonadida	Protaspa-lineage	Protaspa-lineage_X	Protaspa-lineage_X_sp.
	Cercozoa	Filosa-Thecofilosea	Cryomonadida	Rhogostoma-lineage		
	Cercozoa	Filosa-Thecofilosea	Cryomonadida	Rhogostoma-lineage	Rhogostoma-lineage_X	Rhogostoma-lineage_X_sp.
	Cercozoa	Filosa-Thecofilosea	Filosa-Thecofilosea_X	Mataza-lineage	Mataza-lineage_X	Mataza-lineage_X_sp.
	Cercozoa	Novel-clade-10-12	Novel-clade-10	Novel-clade-10_X	Novel-clade-10_XX	Novel-clade-10_XX_sp.
	Cercozoa	Novel-clade-10-12	Tremulida	Tremulidae	Tremula	
	Cercozoa	Novel-clade-10-12	Tremulida	Tremulidae	Tremula	Tremula_sp.
Stramenopiles	Stramenopiles_X	Bicoecea	Bicoecales	Bicoecaceae	Bicoecaceae_X	Bicoecaceae_X_sp.
	Stramenopiles_X	Bicoecea	Bicoecales	Bicoecaceae		
	Stramenopiles_X	Bicoecea	Pseudodendromonadales	Pseudodendromonadales_X		
	Stramenopiles_X	Bicoecea	Pseudodendromonadales	Pseudodendromonadales_X	Paramonas	Paramonas_globosa
	Stramenopiles_X	Bicoecea	Pseudodendromonadales	Pseudodendromonadales_X	Pseudodendromonadales_XX	Pseudodendromonadales_XX_sp.
	Stramenopiles_X	Labyrinthulea	Thraustochytriales	Thraustochytriaceae	Amphifilidae	Amphifilidae_sp.

	Stramenopiles_X	Labyrinthulea	Thraustochytriales	Thraustochytriaceae		
	Stramenopiles_X	Labyrinthulea	Thraustochytriales	Thraustochytriaceae	Thraustochytriaceae_X	Thraustochytriaceae_X_sp.
	Stramenopiles_X	MAST	MAST-12	MAST-12_X	MAST-12_XX	MAST-12_XX_sp.
	Stramenopiles_X	MAST	MAST-12	MAST-12A	MAST-12A_X	MAST-12A_X_sp.
	Stramenopiles_X	MAST	MAST-12	MAST-12C	MAST-12C_X	MAST-12C_X_sp.
	Stramenopiles_X	MAST	MAST-2	MAST-2A	MAST-2A_X	MAST-2A_X_sp.
	Stramenopiles_X	MAST	MAST-3	MAST-3J	MAST-3J_X	MAST-3J_X_sp.
	Stramenopiles_X	Oomycota	Oomycota_X	Haptoglossales	Haptoglossa	
	Stramenopiles_X	Oomycota	Oomycota_X	Oomycota_XX	Oomycota_XXX	Oomycota_XXX_sp.
	Stramenopiles_X	Oomycota	Oomycota_X	Peronosporales		
	Stramenopiles_X	Oomycota	Oomycota_X	Peronosporales	Peronosporales_X	Peronosporales_X_sp.
	Stramenopiles_X	Oomycota	Oomycota_X	Peronosporales	Pythiaceae	Pythiaceae_sp.
	Stramenopiles_X	Oomycota	Oomycota_X	Peronosporales	Pythium	
	Stramenopiles_X	Oomycota	Oomycota_X	Peronosporales	Pythium	Pythium_adhaerens
	Stramenopiles_X	Oomycota	Oomycota_X	Saprolegniales	Aphanomyces	Aphanomyces_sp.
	Stramenopiles_X	Oomycota	Oomycota_X	Saprolegniales		
	Stramenopiles_X	Pirsonia_Clade	Pirsonia_Clade_X	Pirsonia_Clade_XX	Pirsonia	Pirsonia_verrucosa
	Stramenopiles_X	Pirsonia_Clade	Pirsonia_Clade_X	Pirsonia_Clade_XX	Pirsonia_Clade__XXX	Pirsonia_Clade__XXX_sp.
	Stramenopiles_X	Stramenopiles_XX	Stramenopiles_XXX	Stramenopiles_XXXX	Stramenopiles_XXXXX	Stramenopiles_XXXXX_sp.
	Stramenopiles_X					

B) Protists ("Phytoplankton")

**Supergroup**	**Division**	**Class**	**Order**	**Family**	**Genus**	**Species**
Alveolata	Dinoflagellata	Dinophyceae	Dinophyceae_X	Tovelliaceae	Tovellia	Tovellia_aveirensis (Tovellia/Woloszynskia)
	Dinoflagellata	Dinophyceae	Gonyaulacales	Ceratiaceae	Ceratium*	Ceratium_furcoides
	Dinoflagellata	Dinophyceae	Gonyaulacales	Ceratiaceae	Ceratium*	Ceratium_hirundinella
	Dinoflagellata	Dinophyceae	Gymnodiniales	Gymnodiniaceae	Gymnodinium*	Gymnodinium_sp.
	Dinoflagellata	Dinophyceae	Gymnodiniales	Gymnodiniaceae	Gyrodinium*	Gyrodinium_helveticum
	Dinoflagellata	Dinophyceae	Peridiniales	Peridiniaceae	Peridinium*	Peridinium_cinctum
	Dinoflagellata	Dinophyceae	Peridiniales	Peridiniaceae	Peridinium*	Peridinium_willei
	Dinoflagellata	Dinophyceae	Peridiniales	Peridiniopsidaceae	Parvodinium	Parvodinium_inconspicuum
	Dinoflagellata	Dinophyceae	Peridiniales	Thoracosphaeraceae		
	Dinoflagellata	Dinophyceae	Peridiniales	Thoracosphaeraceae	Scrippsiella	Scrippsiella_acuminata
	Dinoflagellata	Dinophyceae	Peridiniales	Thoracosphaeraceae	Thoracosphaeraceae_X	Thoracosphaeraceae_X_sp.
	Dinoflagellata	Dinophyceae	Prorocentrales	Prorocentraceae	Prorocentrum	Prorocentrum_sp.
	Dinoflagellata	Dinophyceae	Suessiales	Borghiellaceae	Baldinia*	Baldinia_sp.
	Dinoflagellata	Dinophyceae	Suessiales	Borghiellaceae		
	Dinoflagellata	Dinophyceae	Suessiales	Suessiaceae	Asulcocephalium	Asulcocephalium_miricentonis
	Dinoflagellata	Dinophyceae	Suessiales	Suessiaceae		
Archaeplastida	Chlorophyta	Chlorodendrophyceae	Chlorodendrales	Chlorodendraceae	Tetraselmis*	Tetraselmis_cordiformis
	Chlorophyta	Chlorophyceae	Chlamydomonadales	Chlamydomonadales_X	Carteria*	
	Chlorophyta	Chlorophyceae	Chlamydomonadales	Chlamydomonadales_X	Chlamydomonadales_XX	Chlamydomonadales_XX_sp.
	Chlorophyta	Chlorophyceae	Chlamydomonadales	Chlamydomonadales_X	Chlamydomonas*	Chlamydomonas_reinhardtii
	Chlorophyta	Chlorophyceae	Chlamydomonadales	Chlamydomonadales_X	Chlamydomonas*	Chlamydomonas_sordida
	Chlorophyta	Chlorophyceae	Chlamydomonadales	Chlamydomonadales_X	Chlamydomonas*	
	Chlorophyta	Chlorophyceae	Chlamydomonadales	Chlamydomonadales_X	Deasonia	Deasonia_sp.
	Chlorophyta	Chlorophyceae	Chlamydomonadales	Chlamydomonadales_X		
	Chlorophyta	Chlorophyceae	Chlamydomonadales	Chlamydomonadales_X	Phacotus	Phacotus_lenticularis
	Chlorophyta	Chlorophyceae	Sphaeropleales	Sphaeropleales_X	Desmodesmus(*)	
	Chlorophyta	Chlorophyceae	Sphaeropleales	Sphaeropleales_X	Hariotina*	Hariotina_reticulata
	Chlorophyta	Chlorophyceae	Sphaeropleales	Sphaeropleales_X	Mychonastes	Mychonastes_sp.
	Chlorophyta	Chlorophyceae	Sphaeropleales	Sphaeropleales_X	Mychonastes	
	Chlorophyta	Chlorophyceae	Sphaeropleales	Sphaeropleales_X		
	Chlorophyta	Chlorophyceae	Sphaeropleales	Sphaeropleales_X	Radiococcus	
	Chlorophyta	Mamiellophyceae	Dolichomastigales	Crustomastigaceae	Crustomastigaceae-AB	Crustomastigaceae-AB_sp.
	Chlorophyta	Mamiellophyceae	Dolichomastigales	Crustomastigaceae		
	Chlorophyta	Mamiellophyceae	Dolichomastigales	Dolichomastigaceae		
	Chlorophyta	Mamiellophyceae	Monomastigales	Monomastigaceae	Monomastigaceae_X	Monomastigaceae_X_sp.
	Chlorophyta	Trebouxiophyceae	Chlorellales	Chlorellales_X	Chlorella	
	Chlorophyta	Trebouxiophyceae	Chlorellales	Chlorellales_X	Chlorellales_XX	Chlorellales_XX_sp.
	Chlorophyta	Trebouxiophyceae	Chlorellales	Chlorellales_X		
	Chlorophyta	Trebouxiophyceae	Chlorellales	Chlorellales_X	Oocystaceae*	Oocystaceae_sp.
	Chlorophyta	Trebouxiophyceae	Trebouxiophyceae_X	Trebouxiophyceae_XX	Botryococcus*	Botryococcus_braunii
	Chlorophyta	Trebouxiophyceae	Trebouxiophyceae_X	Trebouxiophyceae_XX	Choricystis	Choricystis_sp.
	Rhodophyta	Bangiophyceae	Cyanidiales	Cyanidiales_X	Cyanidiales_XX	Cyanidiales_XX_sp.
	Streptophyta	Zygnemophyceae	Zygnemophyceae_X	Zygnemophyceae_XX	Closterium*	
	Streptophyta	Zygnemophyceae	Zygnemophyceae_X	Zygnemophyceae_XX	Mougeotia*	Mougeotia_sp.
	Streptophyta	Zygnemophyceae	Zygnemophyceae_X	Zygnemophyceae_XX		
	Streptophyta	Zygnemophyceae	Zygnemophyceae_X	Zygnemophyceae_XX	Spirotaenia	
	Streptophyta	Zygnemophyceae	Zygnemophyceae_X	Zygnemophyceae_XX	Spirotaenia	Spirotaenia_alpina
	Streptophyta	Zygnemophyceae	Zygnemophyceae_X	Zygnemophyceae_XX	Staurastrum*	Staurastrum_punctulatum
	Streptophyta	Zygnemophyceae	Zygnemophyceae_X	Zygnemophyceae_XX	Staurastrum*	Staurastrum_sp.
Hacrobia	Cryptophyta	Cryptophyceae	Cryptophyceae_X	Basal_Cryptophyceae-1	Basal_Cryptophyceae-1_X	Basal_Cryptophyceae-1_X_sp.
	Cryptophyta	Cryptophyceae	Cryptophyceae_X	Cryptomonadales	Cryptomonadales_X	Cryptomonadales_X_sp.
	Cryptophyta	Cryptophyceae	Cryptophyceae_X	Cryptomonadales	Cryptomonas*	Cryptomonas_curvata
	Cryptophyta	Cryptophyceae	Cryptophyceae_X	Cryptomonadales	Cryptomonas*	Cryptomonas_tetrapyrenoidosa
	Cryptophyta	Cryptophyceae	Cryptophyceae_X	Cryptomonadales	Cryptomonas*	
	Cryptophyta	Cryptophyceae	Cryptophyceae_X	Cryptomonadales		
	Cryptophyta	Cryptophyceae	Cryptophyceae_X	Cryptomonadales	Plagioselmis*	
	Haptophyta	Prymnesiophyceae	Prymnesiales	Chrysochromulinaceae	Chrysochromulina*	Chrysochromulina_parva
	Haptophyta	Prymnesiophyceae	Prymnesiales	Chrysochromulinaceae	Chrysochromulina*	Chrysochromulina_sp.
	Katablepharidophyta	Katablepharidaceae	Katablepharidales	Katablepharidales_X	Katablepharidales_XX	Katablepharidales_XX_sp.
Stramenopiles	Ochrophyta	Bacillariophyta	Bacillariophyta_X	Araphid-pennate	Asterionella*	Asterionella_formosa
	Ochrophyta	Bacillariophyta	Bacillariophyta_X	Araphid-pennate	Fragilaria*	Fragilaria_bidens
	Ochrophyta	Bacillariophyta	Bacillariophyta_X	Araphid-pennate	Fragilaria*	Fragilaria_crotonensis
	Ochrophyta	Bacillariophyta	Bacillariophyta_X	Araphid-pennate	Fragilaria*	
	Ochrophyta	Bacillariophyta	Bacillariophyta_X	Araphid-pennate	Synedra*	
	Ochrophyta	Bacillariophyta	Bacillariophyta_X	Araphid-pennate	Synedra*	Synedra_acus
	Ochrophyta	Bacillariophyta	Bacillariophyta_X	Polar-centric-Mediophyceae	Discostella(*)	Discostella_sp.
	Ochrophyta	Bacillariophyta	Bacillariophyta_X	Polar-centric-Mediophyceae		
	Ochrophyta	Bacillariophyta	Bacillariophyta_X	Polar-centric-Mediophyceae	Stephanodiscus*	
	Ochrophyta	Bacillariophyta	Bacillariophyta_X	Radial-centric-basal-Coscinodiscophyceae	Aulacoseira*	Aulacoseira_granulata
	Ochrophyta	Bacillariophyta	Bacillariophyta_X	Radial-centric-basal-Coscinodiscophyceae	Aulacoseira*	Aulacoseira_islandica
	Ochrophyta	Bacillariophyta	Bacillariophyta_X	Radial-centric-basal-Coscinodiscophyceae	Melosira*	Melosira_varians
	Ochrophyta	Bolidophyceae	Parmales	Parmales_env_2	Parmales_env_2_X	Parmales_env_2_X_sp.
	Ochrophyta	Bolidophyceae	Parmales	Parmales_X	Parmales_XX	Parmales_XX_sp.
	Ochrophyta	Chrysophyceae	Chrysophyceae_X	Chrysophyceae_Clade_C	Ochromonas*	
	Ochrophyta	Chrysophyceae	Chrysophyceae_X	Chrysophyceae_Clade_C	Ochromonas*	Ochromonas_sp.
	Ochrophyta	Chrysophyceae	Chrysophyceae_X	Chrysophyceae_Clade-B1	Chrysophyceae_Clade-B1_X	Chrysophyceae_Clade-B1_X_sp.
	Ochrophyta	Chrysophyceae	Chrysophyceae_X	Chrysophyceae_Clade-B2	Chrysosphaerella	
	Ochrophyta	Chrysophyceae	Chrysophyceae_X	Chrysophyceae_Clade-B2		

	Ochrophyta	Chrysophyceae	Chrysophyceae_X	Chrysophyceae_Clade-C	Chrysophyceae_Clade-C_X	Chrysophyceae_Clade-C_X_sp.
	Ochrophyta	Chrysophyceae	Chrysophyceae_X	Chrysophyceae_Clade-C	Dinobryon*	Dinobryon_divergens
	Ochrophyta	Chrysophyceae	Chrysophyceae_X	Chrysophyceae_Clade-C	Dinobryon*	Dinobryon_sociale
	Ochrophyta	Chrysophyceae	Chrysophyceae_X	Chrysophyceae_Clade-C		
	Ochrophyta	Chrysophyceae	Chrysophyceae_X	Chrysophyceae_Clade-C	Poteriospumella	Poteriospumella_lacustris
	Ochrophyta	Chrysophyceae	Chrysophyceae_X	Chrysophyceae_Clade-C	Uroglena*	
	Ochrophyta	Chrysophyceae	Chrysophyceae_X	Chrysophyceae_Clade-C	Uroglena*	Uroglena_americana
	Ochrophyta	Chrysophyceae	Chrysophyceae_X	Chrysophyceae_Clade-E	Chrysophyceae_Clade-E_X	Chrysophyceae_Clade-E_X_sp.
	Ochrophyta	Chrysophyceae	Chrysophyceae_X	Chrysophyceae_Clade-E		
	Ochrophyta	Chrysophyceae	Chrysophyceae_X	Chrysophyceae_Clade-F	Chrysophyceae_Clade-F_X	Chrysophyceae_Clade-F_X_sp.
	Ochrophyta	Chrysophyceae	Chrysophyceae_X	Chrysophyceae_Clade-F		
	Ochrophyta	Chrysophyceae	Chrysophyceae_X	Chrysophyceae_Clade-F	Paraphysomonas	Paraphysomonas_sp.
	Ochrophyta	Chrysophyceae	Chrysophyceae_X	Chrysophyceae_Clade-F	Paraphysomonas	Paraphysomonas_vestita
	Ochrophyta	Chrysophyceae	Chrysophyceae_X	Chrysophyceae_Clade-G	Chrysophyceae_Clade-G_X	Chrysophyceae_Clade-G_X_sp.
	Ochrophyta	Chrysophyceae	Chrysophyceae_X	Chrysophyceae_Clade-H	Chrysophyceae_Clade-H_X	Chrysophyceae_Clade-H_X_sp.
	Ochrophyta	Chrysophyceae	Chrysophyceae_X	Chrysophyceae_XX	Chrysophyceae_XXX	Chrysophyceae_XXX_sp.
	Ochrophyta	Dictyochophyceae	Dictyochophyceae_X	Pedinellales		
	Ochrophyta	Dictyochophyceae	Dictyochophyceae_X	Pedinellales	Pedinellales_X	Pedinellales_X_sp.
	Ochrophyta	Dictyochophyceae	Dictyochophyceae_X	Pedinellales	Pseudopedinella	Pseudopedinella_sp.
	Ochrophyta	Eustigmatophyceae	Eustigmatophyceae_X	Eustigmatophyceae_XX		
	Ochrophyta	Eustigmatophyceae	Eustigmatophyceae_X	Eustigmatophyceae_XX	Nannochloropsis	Nannochloropsis_granulata
	Ochrophyta	Synurophyceae	Synurales	Synurales_X	Mallomonas*	Mallomonas_tonsurata
	Ochrophyta	Synurophyceae	Synurales	Synurales_X	Mallomonas*	
	Ochrophyta	Synurophyceae	Synurales	Synurales_X	Synurales_XX	Synurales_XX_sp.
	Ochrophyta	Xanthophyceae	Xanthophyceae_X	Xanthophyceae_XX	Tribonema*	Tribonema_aequale
	Ochrophyta					

C) Fungi

**Supergroup**	**Division**	**Class**	**Order**	**Family**	**Genus**	**Species**
Opisthokonta	Fungi	Ascomycota	Pezizomycotina	Sordariomycetes	Lecanicillium	
	Fungi	Ascomycota	Pezizomycotina	Sordariomycetes		
	Fungi	Ascomycota	Saccharomycotina	Saccharomycetales	Cyberlindnera	Cyberlindnera_jadinii
	Fungi	Ascomycota	Saccharomycotina	Saccharomycetales	Meyerozyma	Meyerozyma_guilliermondii
	Fungi	Basidiomycota	Agaricomycotina	Tremellomycetes	Cryptococcus	Cryptococcus_carnescens
	Fungi	Basidiomycota	Ustilaginomycotina	Exobasidiomycetes	Tilletiopsis	Tilletiopsis_pallescens
	Fungi	Blastocladiomycota	Blastocladiomycotina	Blastocladiomycetes	Hyaloraphidium	Hyaloraphidium_curvatum
	Fungi	Chytridiomycota	Chytridiomycotina	Chytridiomycetes	Avachytrium	Avachytrium_platense
	Fungi	Chytridiomycota	Chytridiomycotina	Chytridiomycetes	Chytridiomycetes_X	Chytridiomycetes_X_sp.
	Fungi	Chytridiomycota	Chytridiomycotina	Chytridiomycetes	Chytridium	Chytridium_olla
	Fungi	Chytridiomycota	Chytridiomycotina	Chytridiomycetes	Chytriomyces	
	Fungi	Chytridiomycota	Chytridiomycotina	Chytridiomycetes		
	Fungi	Chytridiomycota	Chytridiomycotina	Chytridiomycetes	Rhizophydium	Rhizophydium_chlorogonii
	Fungi	Chytridiomycota	Chytridiomycotina	Chytridiomycetes	Rhizophydium	Rhizophydium_sp.
	Fungi	Chytridiomycota	Chytridiomycotina	Chytridiomycetes	Rhyzophidiales_X	
	Fungi	Chytridiomycota	Chytridiomycotina	Chytridiomycetes	Rhyzophidiales_X	Rhyzophidiales_X_sp.
	Fungi	Chytridiomycota	Chytridiomycotina	Chytridiomycetes	Rhyzophidiales_X	Rhyzophidiales_XX_sp.
	Fungi	Chytridiomycota	Chytridiomycotina	Chytridiomycetes	Spizellomycetales-and-Rhizophlyctidales	Spizellomycetales-and-Rhizophlyctidales_sp.
	Fungi	Chytridiomycota	Chytridiomycotina	Chytridiomycetes	Zygorhizidium	Zygorhizidium_sp.
	Fungi	Chytridiomycota	Chytridiomycotina	Chytridiomycotina_X	Chytridiomycotina_XX	Chytridiomycotina_XX_sp.
	Fungi	Cryptomycota	Cryptomycotina	Cryptomycotina_X	Cryptomycotina_XX	Cryptomycotina_XX_sp.
	Fungi	Cryptomycota	Cryptomycotina	Cryptomycotina_X		
	Fungi	Cryptomycota	Cryptomycotina	Cryptomycotina_X	Rozella	Rozella_allomycis
	Fungi	Fungi_X	Fungi_XX	Fungi_XXX	Fungi_XXXX	Fungi_XXXX_sp.
