# Supplementary material for: Unraveling the Diversity of Eukaryotic Microplankton in a Large and Deep Perialpine Lake Using a High Throughput Sequencing Approach
Source: Front Microbiol. 2020 May 7;11:789. doi: 10.3389/fmicb.2020.00789 (PMC7221148; doi:10.3389/fmicb.2020.00789)
Supplement: Supplementary file 9 [file Data_Sheet_2.PDF]

Supplementary Table 2 First 40 dominant microeukaryotes in Lake Garda; only taxa identified to the genus level following the classification criteria by PR^2 (Guillou et al., 2013) were included. For each division, taxa are ordered by the relative abundance of reads (% dom.) computed on the whole set of samples. All the listed genera showed a BLAST identity of the more abundant ASVs higher than at least 95%, with the exclusion of a few more broad classifications, i.e. Strobilidiidae_X and Perkinsida_XXX (ca. 93%) and MAST-12C_X (> 90%). ASVs indicates the number of amplicon sequence variants per genus. % seq_sim reports the mean percentage DNA base similarity among ASVs.

**Division**	**Class**	**Order**	**Family**	**Genus**	**% dom.**	**ASVs**	**% seq_sim**
Ciliophora	CONThreeP	CONThreeP_X	CONThreeP_XX	Askenasia	4.5	24	92.1
	Spirotrichea	Choreotrichida	Strobilidiidae_D	Rimostrombidium_D	2.9	1	
	Oligohymenophorea	Scuticociliatia_2	Histiobalantiidae	Histiobalantium	1.9	3	93.1
	Spirotrichea	Strombidiida	Pelagostrombidiidae	Limnostrombidium	1.6	2	99.5
	Spirotrichea	Choreotrichida	Strobilidiidae	Strobilidiidae_X	1.4	1	
	Spirotrichea	Choreotrichida	Strobilidiidae_A	Rimostrombidium_A	1.4	1	
	Spirotrichea	Choreotrichida	Strobilidiidae_B	Strobilidiidae_B_X	1.0	1	
	Spirotrichea	Hypotrichia	Halteriidae	Halteriidae_X	0.9	1	
	Spirotrichea	Choreotrichida	Strobilidiidae_J	Strobilidiidae_J_X	0.5	1	
	Spirotrichea	Strombidiida_A	Strombidiida_A_X	Strombidiida_A_XX	0.5	3	98.0
	Oligohymenophorea	Peritrichia_2	Sessilida	Vorticella	0.4	6	91.7
	Spirotrichea	Tintinnida	Tintinnidiidae	Tintinnidium	0.3	2	92.7
Dinoflagellata	Dinophyceae	Gymnodiniales	Gymnodiniaceae	Gyrodinium	1.9	1	
	Dinophyceae	Gonyaulacales	Ceratiaceae	Ceratium	1.9	5	95.9
	Dinophyceae	Suessiales	Suessiaceae	Asulcocephalium	0.8	9	97.6
Perkinsea	Perkinsida	Perkinsida_X	Perkinsida_XX	Perkinsida_XXX	2.1	30	92.2
Chlorophyta	Chlorodendrophyceae	Chlorodendrales	Chlorodendraceae	Tetraselmis	1.3	5	99.0
	Chlorophyceae	Chlamydomonadales	Chlamydomonadales_X	Chlamydomonas	0.9	5	92.5
	Chlorophyceae	Sphaeropleales	Sphaeropleales_X	Mychonastes	0.4	4	98.6
Streptophyta	Zygnemophyceae	Zygnemophyceae_X	Zygnemophyceae_XX	Closterium	1.6	3	94.9
	Zygnemophyceae	Zygnemophyceae_X	Zygnemophyceae_XX	Mougeotia	1.0	4	96.7
Cryptophyta	Cryptophyceae	Cryptophyceae_X	Cryptomonadales	Cryptomonas	10.4	4	97.3
	Cryptophyceae	Cryptophyceae_X	Cryptomonadales	Plagioselmis	9.3	1	
	Cryptophyceae	Cryptophyceae_X	Basal_Cryptophyceae-1	Basal_Cryptophyceae-1_X	1.8	10	97.7
Katablepharidophyta	Katablepharidaceae	Katablepharidales	Katablepharidales_X	Katablepharidales_XX	2.6	6	97.5
Telonemia	Telonemia_X	Telonemia_XX	Telonemia-Group-2	Telonemia-Group-2_X	2.3	6	98.7
Fungi	Chytridiomycota	Chytridiomycotina	Chytridiomycetes	Rhyzophidiales_X	1.7	26	89.8
Cercozoa	Filosa-Imbricatea	Filosa-Imbricatea_X	Novel-clade-2	Novel-clade-2_X	2.9	9	91.2
	Filosa-Thecofilosea	Cryomonadida	Protaspa-lineage	Protaspa-lineage_X	0.5	7	97.6
	Novel-clade-10-12	Novel-clade-10	Novel-clade-10_X	Novel-clade-10_XX	0.5	18	90.8
Ochrophyta	Chrysophyceae	Chrysophyceae_X	Chrysophyceae_Clade-C	Uroglena	2.9	4	99.6
	Chrysophyceae	Chrysophyceae_X	Chrysophyceae_Clade-E	Chrysophyceae_Clade-E_X	2.3	12	95.7
	Bacillariophyta	Bacillariophyta_X	Polar-centric-Mediophyceae	Stephanodiscus	1.4	1	
	Chrysophyceae	Chrysophyceae_X	Chrysophyceae_XX	Chrysophyceae_XXX	0.8	14	93.2
	Bacillariophyta	Bacillariophyta_X	Araphid-pennate	Fragilaria	0.7	7	96.8
	Synurophyceae	Synurales	Synurales_X	Synurales_XX	0.6	14	96.5
	Bacillariophyta	Bacillariophyta_X	Radial-centric-basal-Coscinodiscophyceae	Aulacoseira	0.5	2	94.5
Stramenopiles_X	Bicoecea	Pseudodendromonadales	Pseudodendromonadales_X	Pseudodendromonadales_XX	1.0	50	87.6
	MAST	MAST-12	MAST-12C	MAST-12C_X	0.4	7	93.9
	MAST	MAST-2	MAST-2A	MAST-2A_X	0.3	1	
