# Supplementary material for: Unraveling the Diversity of Eukaryotic Microplankton in a Large and Deep Perialpine Lake Using a High Throughput Sequencing Approach
Source: Front Microbiol. 2020 May 7;11:789. doi: 10.3389/fmicb.2020.00789 (PMC7221148; doi:10.3389/fmicb.2020.00789)
Supplement: Supplementary file 10 [file Data_Sheet_3.PDF]

Supplementary Table 3 (A), left panel, Spearman correlation coefficients between % phytoplankton biovolumes estimated by light microscopy and % HTS reads and, right panel, quantile regressions (τ=0.50) between % phytoplankton biovolume (y) and % HTS reads (x). (B), as in (A), but with computations using phytoplankton densities. SlopeQ50, regression slope; SE, standard error. Algal taxa are defined as in Guiry and Guiry (2020), following the classification system used in light microscopy; under "Taxon level" the suffixes "_o" and "_f" indicate Order and Family, respectively; [(1)] Komárek and Fott (1983); [(2)] Wehr and Sheath (2003). Significance codes, *P*: *** ≤0.001; ** ≤0.01; * ≤0.05; + ≤0.10; ns >0.10.

(A)

Phylum	Taxon level	Biovolume vs HTS Spearman	*P*	Biovolume vs HTS SlopeQ50 (SE)	*P*
Chlorophyta	Chlorococcales[(1)]	0.47	***	0.21 (0.10)	*
	Chlamydomonadales_o	0.01	ns	-0.01 (0.15)	ns
Charophyta	Desmidiales_o	0.79	***	0.45 (0.22)	*
	Zygnematales_o	0.84	***	2.44 (0.57)	***
Ochrophyta	Chromulinales_o	0.39	**	0.47 (0.24)	*
	Synurales_o	0.16	ns	0.24 (0.12)	+
	Tribonematales_o	0.29	*	1.06 (1.29)	ns
Bacillariophyta	Centric[(2)]	0.80	***	1.14 (0.12)	***
	Pennate[(2)]	0.74	***	15.1 (2.51)	***
Miozoa	Ceratiaceae_f	0.55	***	2.39 (0.42)	***
	Gyrodiniaceae_f	0.48	***	0.45 (0.10)	***
	Peridiniaceae_f	0.30	*	3.08 (1.47)	*

(B)

Phylum	Taxon level	Density vs HTS Spearman	*P*	Density vs HTS SlopeQ50 (SE)	*P*
Chlorophyta	Chlorococcales[(1)]	0.46	***	1.26 (0.50)	*
	Chlamydomonadales_o	0.10	ns	0.004 (0.06)	ns
Charophyta	Desmidiales_o	0.82	***	0.17 (0.02)	***
	Zygnematales_o	0.85	***	0.57 (0.24)	*
Ochrophyta	Chromulinales_o	0.37	**	0.66 (0.31)	*
	Synurales_o	0.29	*	0.11 (0.07)	ns
	Tribonematales_o	0.30	*	2.49 (1.98)	ns
Bacillariophyta	Centric[(2)]	0.46	***	0.60 (0.14)	***
	Pennate[(2)]	0.75	***	9.80 (1.42)	***
Miozoa	Ceratiaceae_f	0.53	***	0.02 (0.01)	*
	Gyrodiniaceae_f	0.60	***	0.03 (0.01)	***
	Peridiniaceae_f	0.28	*	0.25 (0.13)	+

References

Guiry, M. D., and Guiry, G. . (2020). Algaebase: Listing the World's Algae. Available at: https://www.algaebase.org/ [Accessed January 31, 2020].

Komárek, J., and Fott, B. (1983). "Chlorophyceae Chlorococcales.," in *Das Phytoplankon des Süßwassers Systematik und Biologie.*, ed. G. Huber–Pestalozzi (Stuttgart: Schweizerbart'sche Verlagsbuchhandlung), 1043.

Wehr, J. D., and Sheath, R. G. (2003). *Freshwater Algae of North America - Ecology and Classification*. Academic Press, Elsevier.
